# How to link theory and experiment for single-chain magnets beyond the Ising model: magnetic properties modeled from *ab initio* calculations of molecular fragments†
†Electronic supplementary information (ESI) available: Comparison of coupling schemes, extrapolation of the correlation length, correlation length and coupling schemes, magnetic susceptibility of a 1D periodic Ising chain, *ab initio* computational models and single-ion properties, fitting of the magnetic susceptibility, determined magnetic coupling constants *J*_calc_, spin states dependent on the single-ion anisotropy, additional POLY_ANISO results, extrapolation of the magnetic susceptibility, magnetic interchain interactions, basis set information, and decomposition of the calculated magnetic susceptibility. See DOI: 10.1039/c9sc02735a


**DOI:** 10.1039/c9sc02735a

**Published:** 2019-08-19

**Authors:** Michael Böhme, Winfried Plass

**Affiliations:** a Institut für Anorganische und Analytische Chemie , Friedrich-Schiller-Universität Jena , Humboldtstraße 8 , 07743 Jena , Germany . Email: sekr.plass@uni-jena.de ; Fax: +49 3641 948132 ; Tel: +49 3641 948130

## Abstract

Magnetic properties of coordination polymers like single-chain magnets (SCMs) are based on magnetic domains, which are formed due to magnetic exchange between neighboring anisotropic spin centers. However, the computational restrictions imposed by the high level of theory needed for an adequate *ab initio* quantum mechanical treatment on the basis of multi-reference methods for these systems limit the feasibility of such calculations to mononuclear fragments as appropriate structural cutouts for the metal centers along the chains. Hence, results from such calculations describe single-ion properties and cannot be directly correlated with experimental data representing magnetic domains. We present a theoretical approach based on *n*-membered Ising-spin rings with *n* = 3–12, which allows us to simulate magnetic domains and to derive important magnetic properties for SCM compounds. Magnetic exchange, which is not provided by calculations of mononuclear fragments, is obtained by fitting the theoretical magnetic susceptibility against experimental data. The presented approach is tested for cobalt(ii)-based SCMs with three types of repeating sequences, which differ in nuclearity and symmetry. The magnetic parameters derived using the presented approach were found to be in good agreement with the experimental data. Moreover, the energy spectra obtained for the three test cases using the presented approach are characteristic of a deviation of the individual systems from the ideal Ising behavior. An extrapolation technique towards larger systems (*n* > 12) is presented which can provide information on the statistical mean length of the magnetic domains in the three investigated SCM compounds.

## Abstract 2

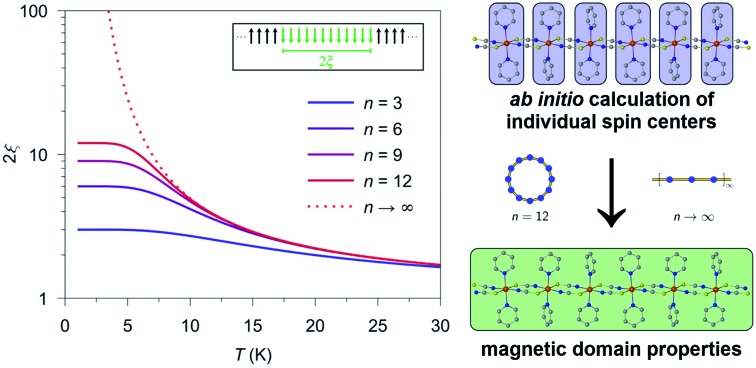
Properties of 1D periodic magnetic chains can be described on the basis of results from *ab initio* multi-reference calculations performed for individual spin centers, which provides a basis for investigations on their dynamic magnetic properties.

## 1 Introduction

Magnetic compounds that show a slow relaxation of magnetization have received a considerable attention in recent years, since they are of great interest for future technologies.^1^–^4^ The so-called single-chain magnets (SCMs) are one-dimensional (1D) coordination polymers and a promising class of nanomagnets.^5^–^7^ Recent reports in the field of SCMs discuss the influence of single-ion anisotropy^8^ and relaxation mechanisms,^9^–^11^ and show interesting properties, *e.g.* the ability of photo-induced SCM behavior.^12^ Over the last two decades, a large variety of SCM compounds have been reported, of which one of the largest groups consists of homometallic cobalt(ii) coordination polymers.^11^,^13^–^24^ This is not surprising, since also a large number of mononuclear cobalt(ii) complexes with a large magnetic anisotropy is known which show a slow relaxation of magnetization.^25^–^35^ In addition, a variety of heterometallic SCM compounds have been reported containing 3d–3d,^36^,^37^ 3d–4d,^38^ and 3d–5d^8^,^39^ transition metal ions. Furthermore, particular attention has also been paid to 3d–4f^40^ and purely lanthanide-based systems due to their large intrinsic anisotropy for some of the trivalent rare-earth metal ions.^41^–^43^

Although a notable number of compounds with SCM behavior have been reported within the last few years, only a few have been investigated by *ab initio* quantum mechanical methods on the level of multi-reference methods.^23^,^24^,^43^–^47^ From our point of view this situation is unsatisfactory, since we are firmly convinced that synergies evolve wherever experimental and theoretical methods can be combined. However, *ab initio* quantum mechanical studies on the multi-reference level for 1D periodic systems like SCMs are challenging and computationally demanding. In general, high-level theoretical calculations are required to adequately describe the complex electronic open-shell structure of paramagnetic centers. Multi-reference methods like CASSCF and CASPT2 are usually employed for such systems, as they include the essential static and dynamic electron correlation. In addition, spin–orbit coupling and relativistic effects are also important and have to be taken into account. Unfortunately, it is currently not feasible to describe larger structural chain fragments or even periodic systems utilizing *ab initio* quantum mechanical methods providing the required high accuracy. As a consequence, coordination polymers need to be divided into smaller structural fragments of individual spin centers, which then can be treated by *ab initio* computational methods.^23^,^24^,^48^ This approach allows us to calculate single-ion properties for the spin centers of SCMs, such as the magnetic axes, corresponding *g* factors, and energies of spin–orbit coupled magnetic states. However, these single-ion parameters cannot be directly related to the experimental magnetic properties of 1D periodic compounds, as they are cooperative in nature and based on magnetic domains.

Herein, we demonstrate how *ab initio* quantum mechanical calculations of mononuclear fragments can be used to determine the magnetic properties of coordination polymers like SCMs. The merit of the presented approach is that it allows for a direct correlation between high-level *ab initio* single-ion properties and experimental data. The basic concept of our approach utilizes the simulation of the magnetic domain properties of 1D periodic compounds in a low temperature range on the basis of *ab initio* calculations of individual mononuclear fragments in combination with an appropriate spin-coupling scheme. The presented approach is used to study the static magnetic properties and offers a first step toward the investigation of dynamic magnetic properties by computational methods. In this work, the theoretical procedure is described and tested on three cobalt(ii)-based 1D chain compounds showing SCM behavior with different topologies. Moreover, it is shown that the possibility of simulating different magnetic domain sizes with our approach can be used to extrapolate magnetic properties for arbitrary domain sizes, which gives a basis to investigate size limits of magnetic domains in real compounds by theory.

## 2 Current state

### 2.1 Single-chain magnets (SCMs)

**Scheme 1:**
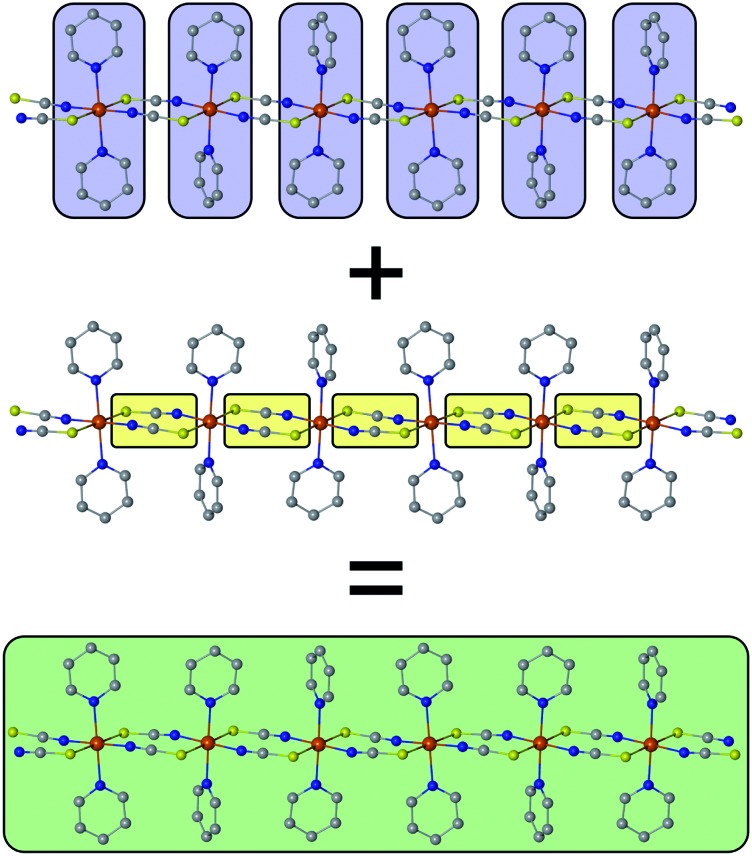
The single-ion anisotropy of the individual spin centers (blue) in combination with the magnetic coupling (yellow) shapes the cooperative magnetic properties of a magnetic domain (green) within a 1D chain (SCM).

The basic concept to design 1D coordination polymers with SCM behavior is illustrated in Scheme 1. Two general prerequisites are mandatory:^5^ (i) paramagnetic spin centers with a high single-ion anisotropy (blue boxes) and (ii) an appropriate exchange coupling between these centers (yellow boxes). Magnetic exchange between the spin centers is required to generate either ferro- or ferrimagnetic ordering along the chain. The latter is a special case where antiferromagnetic exchange between neighboring magnetic ions with different spins leads to a non-vanishing magnetic moment. In this work, however, for the sake of simplicity we will only focus on ferromagnetically coupled chains with an ideal Ising anisotropy. For example, in real compounds, the latter assumption may be considered justifiable when strongly anisotropic metal ions are involved such as cobalt(ii) in a suitable coordination sphere.^8^

The green box in Scheme 1 represents a magnetic domain within a SCM, for which the specific properties are determined by the combination of the individual single-ion anisotropies and magnetic couplings. The properties and size of these magnetic domains depend on several parameters, *e.g.* temperature, magnetic exchange and single-ion anisotropies. Thus, it is essential to investigate the magnetic domains in order to understand the magnetic properties of a SCM. At this point it should be noted that it is far beyond the scope of this work to provide a detailed description of the fundamentals of SCM theory. Therefore, the interested reader is referred to relevant reviews in the literature.^49^–^60^

### 2.2 Computational studies on SCMs

For an *ab initio* quantum mechanical description of a magnetic domain of a specific size (green box in Scheme 1), it would be formally necessary to apply a suitable computational method to a structural model of the same size. However, computational limitations, due to the extremely long computing time and lack of availability of suitable resources, even hamper the treatment of rather small magnetic domains. In addition, it would generally be required to investigate magnetic domains of different sizes by quantum mechanical methods.

Instead, current descriptions of SCMs are based on the application of multi-reference methods such as CASSCF/CASPT2/RASSI-SO to mononuclear fragments, which is due to computational limitations and the lack of suitable methods to describe periodic systems at such a theoretical level. Consequently, the calculated properties based on mononuclear fragments (blue boxes in Scheme 1) can only give a limited insight into the complex magnetic behavior of a SCM compound. Nonetheless, these calculated single-ion properties, *e.g.* the orientation of the magnetic axes, the corresponding *g* factors, and the energies of excited states, can be of great interest for the understanding and design of SCM compounds.

However, the required information on the exchange coupling between the magnetic centers within the chain is not accessible through suitable high level *ab initio* calculations due to technical constraints in terms of hardware and computational limits. As an example, for a simple computational model based on a dinuclear cobalt(ii) fragment a corresponding CASSCF calculation needs to include at least the two 3d valence shells of both cobalt(ii) centers in the active-space as well as an unknown number of relevant orbitals provided by the coordinating and bridging ligand systems. In addition, also other effects like spin–orbit coupling and dynamic correlation can be crucial. As a feasible alternative, the magnetic exchange between individual spin centers (see yellow boxes in Scheme 1) is often determined by DFT methods, such as broken-symmetry DFT (BS-DFT) and constraint DFT (C-DFT).^23^,^24^,^48^ In general, however, it is challenging with such single-determinant approaches to obtain meaningful values in terms of experimental reference as they are limited in their ability to describe complex electronic open-shell structures. Moreover, this directly leads to the intrinsic question of which is the ‘most suitable’ density functional offering the highest accuracy.

This situation prevents the treatment of the magnetic properties of SCMs at a consistent level of quantum mechanical theory. As a result, the low temperature properties of a magnetic domain (green box in Scheme 1) as measured in the experiment cannot be reproduced solely on the basis of *ab initio* calculations for which only the properties of single-ion fragments are accessible (blue boxes in Scheme 1). This raises at least two important questions: (i) how to represent a 1D periodic compound from a theoretical point of view using a finite model in terms of a spin-coupling scheme and (ii) how to determine the missing piece of information, *i.e.* magnetic exchange (yellow boxes in Scheme 1), which is essential for applying any kind of spin-coupling scheme.

## 3 Theoretical background

### 3.1 Magnetic coupling schemes for 1D periodic compounds

At this point, it is necessary to introduce a spin-coupling scheme, which adequately describes 1D periodic chains of Ising spins. It is important to note that a coupling scheme represents a topology which solely describes which pairs of spin centers are magnetically coupled, but does not represent actual geometric structures.

**Scheme 2:**
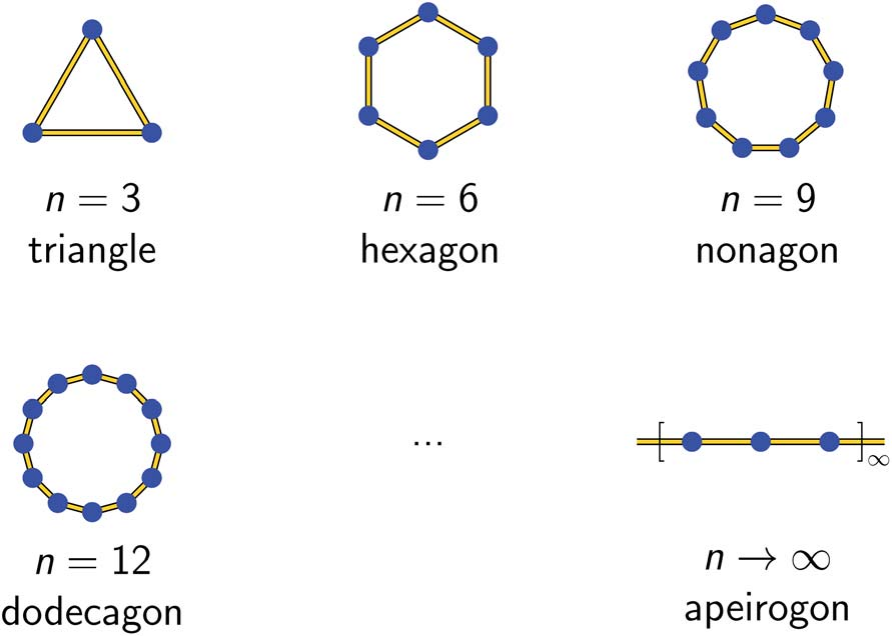
Graphical representation of an ***n*-membered spin ring** coupling scheme for selected model sizes. The blue spheres represent individual spin centers, whereas the yellow lines denote the magnetic exchange interactions between them. The extrapolation *n* → ∞ is indistinguishable from a 1D periodic chain.

A first intuitive representation of 1D periodic chains would be a finite chain fragment with *n* elements (hereafter denoted as ***n*-membered open chain**). The corresponding spin Hamiltonian is given in eqn (1).1
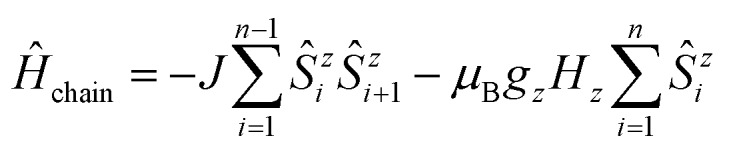
An alternative approach was introduced by Glauber in which he showed that the dynamic process of spin-flips in an Ising chain can be described using a so-called closed *n*-particle ring system (hereafter denoted as ***n*-membered spin ring**).^50^ The spin Hamiltonian for such a system is defined in eqn (2).2
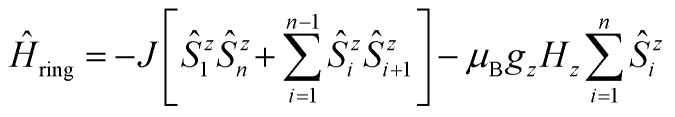
A graphical representation of the coupling scheme of ***n*-membered spin rings** depending on *n* is illustrated in Scheme 2. The scheme also shows that an *n*-membered ring with *n* → ∞, a so-called apeirogon, becomes indistinguishable from 1D periodic chains. Additionally, the coupling scheme of an ***n*-membered open chain** can be interpreted as a special case of Glauber's approach where the magnetic coupling between the first and the *n*-th element was removed.

Figs. S1 and S2† depict the resulting spin multiplets parameterized by *J* for both coupling schemes and selected model sizes *n* as obtained using eqn (1) and (2). This illustrates two fundamental differences between both coupling schemes: (i) the number of obtained spin multiplets and (ii) the energy separation between these multiplets. For the coupling scheme of an ***n*-membered spin ring** the energy separation between consecutive spin multiplets is Δ*E* = |*J*|, which is the exact value for an infinite (periodic) chain of Ising spins (*S*_eff_ = 1/2, *vide infra*). Whereas in the case of a coupling scheme of an ***n*-membered open chain** the energy separation between the individual spin multiplets is Δ*E* = |*J*/2|. Unfortunately, for both coupling schemes the number of spin states rapidly grows with 2^*n*^ and thus limits the maximum model size that can be simulated.

For both presented coupling schemes, a Hamiltonian based on an effective spin model of *S*_eff_ = 1/2 is assumed. It is important to note that this assumed effective spin *S*_eff_ is a theoretical concept and does not represent the real spin *S* of the individual metal centers.^61^ In fact, the effective spin *S*_eff_ describes a fictitious spin such that a specific number of spin states, given by 2*S*_eff_ + 1, is taken into account. Consequently, for isolated Kramers doublets (KDs), an effective *S*_eff_ = 1/2 is assigned. As a result, the coupling constant *J* for an effective spin model of *S*_eff_ = 1/2 is different from the magnetic coupling constant that is related to a model utilizing the actual spin *S*.

### 3.2 Magnetic domains and correlation length

Formally, the low temperature properties of a SCM compound, *e.g.* the static magnetic susceptibility *χ*_M_*T*, clearly differ from the properties of the individual independent spin centers. This phenomenon is based on cooperative interactions, *i.e.* the magnetic coupling between neighboring spin centers, forming magnetic domains. Hence, a prerequisite to describe the magnetic properties of SCM compounds is a basic understanding of the behavior of the magnetic domains.

For a statistical treatment of the nearest-neighbor interactions of an *i*-th spin (*i.e.* with the *i* + 1 and *i* – 1 spin), Glauber introduced the concept of a correlation function *γ*, which is given in eqn (3) for Ising spins (*S*_eff_ = 1/2).^49^,^50^
3
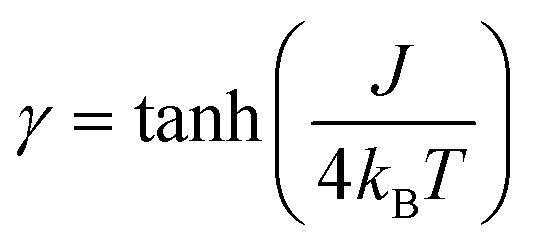
It can be seen from eqn (3) that the correlation function *γ* depends on two quantities: (i) the magnetic coupling *J*, which is a specific property of a SCM compound and is mainly determined by its structure, and (ii) the given temperature.

**Scheme 3:**
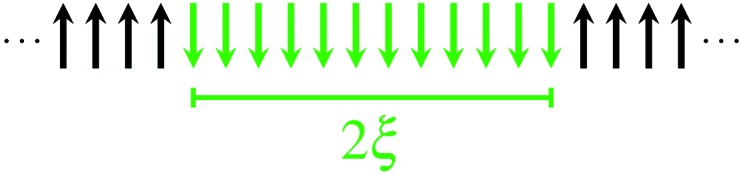
Graphical representation of the correlation length 2*ξ*.

The size of a magnetic domain, which is a statistical mean value, can be expressed by the so-called correlation length 2*ξ* which depends on the correlation function *γ*. Scheme 3 shows a graphical representation of 2*ξ*. For 1D periodic chains of Ising spins (*S*_eff_ = 1/2) the temperature-dependent correlation length 2*ξ*_∞_ is given in eqn (4).^50^,^54^
4
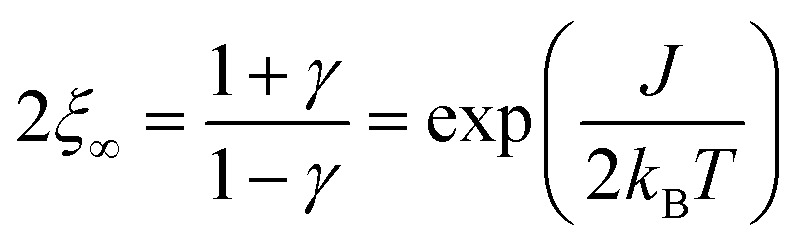



The corresponding expressions for correlation lengths of the two different coupling schemes of an ***n*-membered open chain** (2*ξ*_chain_(*n*)) and an ***n*-membered spin ring** (2*ξ*_ring_(*n*)) are given in eqn (5) and (6), respectively.^62^,^63^
5
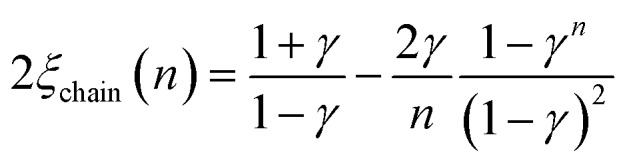

6
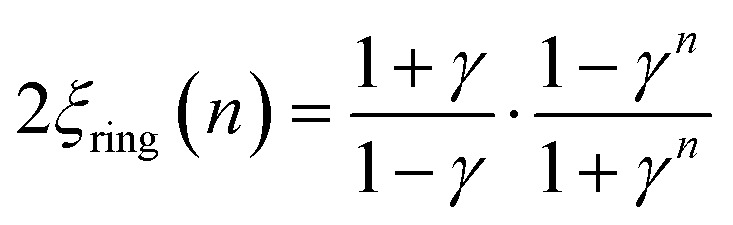
The extrapolation *n* → ∞ for both correlation lengths 2*ξ*_chain_ and 2*ξ*_ring_ is given in eqn (S1) and (S2) (see the section Extrapolation of the correlation length in the ESI†) and reproduces the correlation length for a 1D periodic chain as given in eqn (4).

**Fig. 1:**
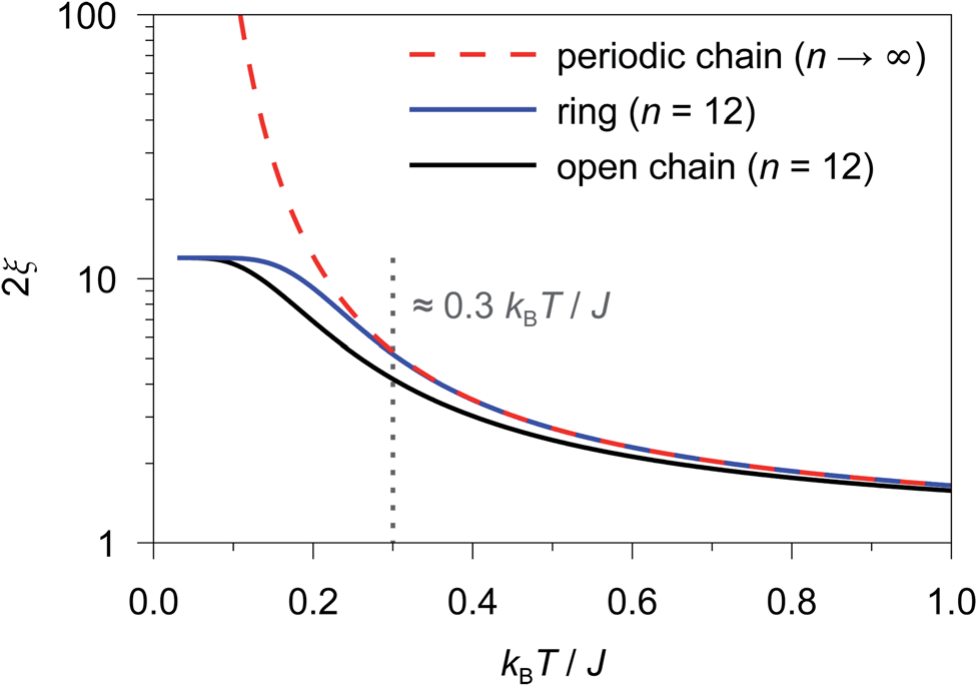
Correlation length as a function of *k*_B_*T*/*J* for a periodic chain (2*ξ*_∞_; red dashed line), a 12-membered spin ring (2*ξ*_ring_(*n* = 12); blue solid line), and a 12-membered open chain (2*ξ*_chain_(*n* = 12); black solid line) of Ising-type spins. The grey dotted line indicates the critical ratio up to which a 12-membered spin ring reproduces the correlation length of a periodic chain (*k*_B_*T*/*J* ≥ 0.3).

As an example, in Fig. 1 the temperature dependence of 2*ξ*_∞_, 2*ξ*_chain_(*n* = 12), and 2*ξ*_ring_(*n* = 12) is depicted assuming a ferromagnetic coupling (*J* > 0). A somewhat different behavior is found for the correlation length 2*ξ*_∞_ of a periodic chain which shows an exponential growth at lower ratios of *k*_B_*T*/*J* as well as singularity at *k*_B_*T*/*J* → 0 (see Fig. 1). However, in real compounds the correlation length is limited (finite-size effect) for a number of reasons (chain defects, impurities, size of the bulk material such as particle size or single-crystal dimensions).^53^ At higher ratios with *k*_B_*T*/*J* ≫ 1 the spin centers become independent of their neighboring spins, and hence the correlation length (correlation function) converges to 2*ξ* ≈ 1 (*γ* ≈ 0).

In the case of the two different coupling schemes of an ***n*-membered open chain** and an ***n*-membered spin ring**, the correlation length at very low temperatures is limited by the model size *n* and reaches 2*ξ* ≈ *n*, which marks the superparamagnetic limit. The correlation length for both coupling schemes as a function of temperature and different numbers of *n* assuming a ferromagnetic coupling of *J*/*k*_B_ = 32 K (also see Table S1†) is depicted in Figs. S3 and S4.†

From Fig. 1 it is obvious that the coupling scheme of a 12-membered spin ring accurately reproduces the temperature dependence of the correlation length of a 1D periodic chain above a certain critical ratio *k*_B_*T*/*J* (see the grey dotted line in Fig. 1). The actual critical ratio *k*_B_*T*/*J* depends on the model size *n* where an increasing model size *n* leads to a decreasing critical ratio. For the given example of a 12-membered spin ring, a lower temperature limit *T*_min_ can be estimated from eqn (7), for which the relation 2*ξ*_∞_ ≈ 2*ξ*_ring_(*n* = 12) is justified.

7 2*ξ*_ring_(*n* = 12)/2*ξ*_∞_ ≥ 0.98 → *T*_min_ ≈ 0.3 *J*/*k*_B_ (*J* > 0)

On the contrary, the coupling scheme of a 12-membered open chain significantly underestimates the correlation length of a periodic chain over a large temperature range (see Fig. 1). Assuming the same model size *n*, the coupling scheme of an ***n*-membered spin ring** is clearly superior to the corresponding open chain scheme. Therefore, for the following discussion, we will solely focus on the coupling scheme of an ***n*-membered spin ring**.

### 3.3 Magnetic susceptibility of 1D periodic Ising chains

The key to study magnetic domains in SCMs (green box in Scheme 1) is the static magnetic susceptibility and its temperature dependence, since the magnetic susceptibility contains information on the single-ion anisotropies and magnetic exchange. The molar magnetic susceptibility *χ*_M_ of an Ising chain can be decomposed into two components, parallel and perpendicular, with respect to the orientation of the spins (eqn (8)).

8 *χ*_M_ = 1/3*χ*_∥_ + 2/3*χ*_⊥_

For a 1D periodic chain of Ising spins, the parallel component of the *χT* product is given in eqn (9).^54^ The product *χ*_∥_*T* is dependent on *g*_∥_ and 2*ξ*_∞_ which correspond to the basic magnetic properties illustrated in Scheme 1, namely the single-ion anisotropy of the individual centers and the magnetic exchange between them.9
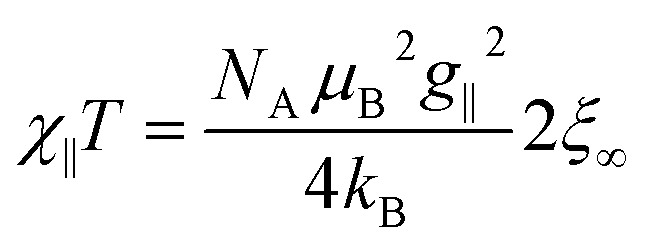



As a consequence of the temperature dependence of 2*ξ*_∞_ (*cf.*eqn (4)), *χ*_∥_*T* shows an exponential increase upon decreasing the temperature assuming *J* > 0 (*i.e. k*_B_*T*/*J* → 0). The situation is fundamentally different for the perpendicular component *χ*_⊥_*T* for an Ising chain which is given in eqn (10).^64^10
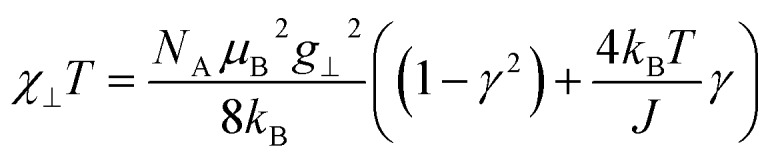



From eqn (10) it becomes evident that *χ*_⊥_ is nearly constant for *k*_B_*T*/*J* < 1 (see Fig. S5† for an example). As a result, the parallel component *χ*_∥_ dominates the magnetic susceptibility in the low temperatures range (*i.e. χ*_M_ ≈ *χ*_∥_/3, see eqn (8)). In the range *k*_B_*T*/*J* < 1 this leads for *χ*_M_ to the approximation given in eqn (11).11
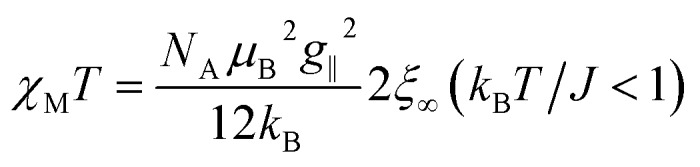



## 4 Methodology

### 4.1 Simulation and fitting of the magnetic susceptibility

For molecular systems, there is an advanced tool within the Molcas package of programs called POLY_ANISO that allows us to determine their magnetic properties from the single-ion properties and the corresponding coupling constants between neighboring spins, both being derived from *ab initio* quantum mechanical calculations.^65^–^67^ Consequently, POLY_ANISO can in principle be used to simulate the magnetic susceptibility of ***n*-membered spin rings** and thus also to address the relevant magnetic properties of magnetic domains in SCMs. Above a critical temperature *T*_min_ (see eqn (7)), this *in silico* simulated magnetic susceptibility would provide a good approximation for the experimental magnetic susceptibility of a 1D periodic chain, because both show an almost perfect agreement for temperature-dependence of the correlation length (see Fig. 1). In this work, we have simulated model sizes of up to *n* = 12 (≡4096 microstates) which marks the maximum feasible limit due to hardware limitations.

A full *ab initio* treatment on the basis of multi-reference methods, however, is hampered by the fact that accurate magnetic coupling constants at this level of theory are currently not available. Therefore, we utilize an alternative approach to link the available experimental and *ab initio* data for SCMs. This is achieved by fitting the simulated magnetic susceptibility to the experimental data *via* the variation of the originally unknown magnetic coupling constants (yellow boxes in Scheme 1). The employed approach treats the magnetic coupling within the Lines model,^68^ which describes the anisotropic exchange interactions using a single parameter *J*_*ij*_ within the basis of the local KDs of the interacting spin centers. This method has been used for molecular systems in the literature.^69^,^70^ The corresponding Hamiltonian is given in eqn (12), where *i* and *j* refer to a coupled spin pair, and *S[combining tilde]*_*i*_ and *S[combining tilde]*_*j*_ are the local effective spin operators associated with the two metal sites *i* and *j*, respectively. Although the Lines model would in general allow for an anisotropic representation of the coupling interaction, this is not appropriate within the current approach, since such a parameterization would not be significant based on the experimental data available.12
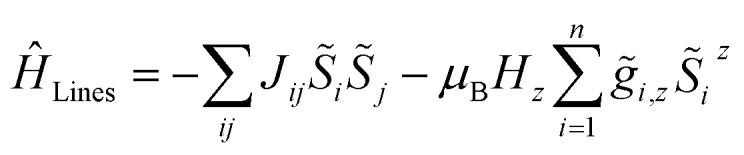



**Scheme 4:**
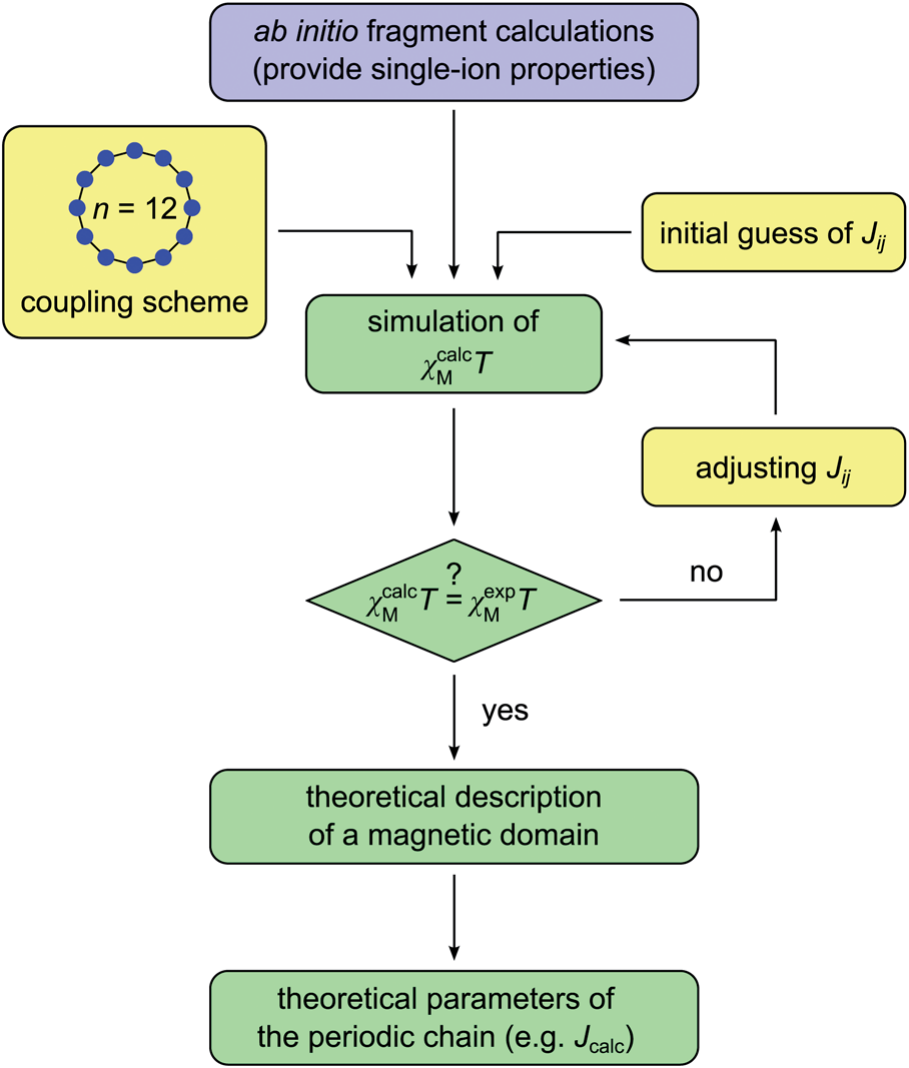
Flow chart for the fitting of the theoretical molar magnetic susceptibility as *χ*calcM*T* against the experimental data *χ*expM*T* (color code: blue – single-ion anisotropy; yellow – magnetic exchange; green – combined properties).

Scheme 4 displays a flow chart which summarizes the overall fitting procedure. The presented coupling scheme of a 12-membered spin ring together with an initial guess for *J*_*ij*_ is used to simulate the magnetic susceptibility based on *ab initio* quantum mechanical calculations of mononuclear fragments. From this the sum of squared residuals is calculated by comparison between the simulated and experimental data. If the simulated *χ*calcM*T* value overestimates (underestimates) the experimental data *χ*expM*T*, the coupling constant *J*_*ij*_ needs to be decreased (increased) by a small amount Δ*J*_*ij*_ for the next iteration cycle. Within the scope of this work, all coupling constants *J*_*ij*_ were fitted to an accuracy of 0.01 K.

It should be noted here that fitting is only appropriate within the specific temperature range (*k*_B_*T*/*J* ≥ 0.3), for which the coupling scheme of a 12-membered spin ring accurately reproduces the correlation length of a 1D periodic chain (see Fig. 1). The actual lower temperature limit *T*_min_ for the fitted range can be estimated using eqn (7). Since *T*_min_ itself depends on *J*, it needs to be verified for the individual case and adjusted if necessary.

### 4.2 Determination of an Ising-like exchange parameter *J*_calc_

The Hamiltonian operators used to describe the exchange interaction between spin centers within the Ising model (see eqn (2)) and the Lines model employed here (see eqn (12)) are based on two different spin representations. As a consequence, the corresponding coupling constants for the Ising and Lines model *J* (*S*_eff_ = 1/2) and *J*_*ij*_ (*S[combining tilde]*_*i*_ and *S[combining tilde]*_*j*_), respectively, are not identical (*i.e. J* ≠ *J*_*ij*_). Nonetheless, it is of great interest to obtain a value from the fitted exchange interaction that can be directly compared with the exchange parameter *J* which is commonly discussed in the context of 1D periodic chains. Unfortunately, in contrast to the Ising model, no simple analytical expression can be given for the resulting spin state energies within the Lines model (see the Hamiltonian in eqn (12)). This is because, although the energies of the spin states scale with *J*_*ij*_, they strongly depend on the local spins *S*_*i*_ and *S*_*j*_, respectively, and in particular on their single-ion anisotropies.

Nevertheless, a link between both spin representations can be established utilizing the energy spectrum of the spin multiplets. The coupling scheme of a 12-membered spin ring with ideal Ising spins (see eqn (2)) leads to seven spin multiplets (Fig. S2†) with an equidistant energy separation of Δ*E* = |*J*|. Similarly, also the simulation of a 12-membered spin ring with the Lines model on the basis of *J*_*ij*_ leads to seven spin multiplets, which, however, are no longer necessarily degenerate, due to non-Ising like single-ion anisotropy of the corresponding metal centers.

For the general case of an ***n*-membered spin ring** with *n* ≥ 3, the corresponding magnetic exchange parameter in terms of an Ising model, which will hereafter be denoted as *J*_calc_, can be defined as the energy gap between the first excited spin multiplet and the ground state doublet (see eqn (13)), both given by the respective mean values (*Ē*).

13 *Ē*_1_ – *Ē*_0_ = *J*_calc_ (*n* ≥ 3)

In the special case of a 12-membered spin ring, *J*_calc_ can also be obtained from the energy difference between the highest spin multiplet and the ground state doublet (see eqn (14)), which is identical to an energy gap of 6|*J*| within an ideal Ising model (see Fig. S2†).

14 *J*_calc_ = (*Ē*_6_ – *Ē*_0_)/6 (*n* = 12)

In addition, the Lines model approach employed within POLY_ANISO allows us to evaluate the magnetic anisotropy of the ***n*-membered spin ring**, used as a model for the 1D periodic chains, in terms of the ***g*** tensor of the ground state doublet. This can then be used for further comparison with relevant experimental data for SCMs, *e.g.* derived by ESR spectroscopy.

### 4.3 Extrapolation of the magnetic susceptibility to *n* > 12

Based on the fitted coupling constants *J*_*ij*_ (see Scheme 4), simulations of the magnetic susceptibility *χ*calcM(*n*)*T* as a function of *n* can be performed using the coupling scheme of an ***n*-membered spin ring** and the fitted *J*_*ij*_ values. The dependence of the magnetic susceptibility *χ*calcM(*n*)*T* on the model size *n* at a given temperature *T* can be studied with the help of these size-dependent simulations. A corresponding fit formula as a function of *n* can be derived on the basis of eqn (4), (6) and (11) and is given in eqn (15).15
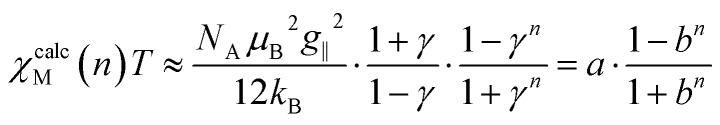



The parameter *a* corresponds to the product *χ*_M_*T* of a periodic chain (*n* → ∞), whereas *b* represents the correlation function (0 < *b* < 1). Formally, the parameters *a* and *b* could be replaced by the functions given in eqn (9) and (3), respectively, both containing the two parameters *J* and *T*. For the fit function in eqn (15), however, the general parameters *a* and *b* were used, as these particularly also allow the treatment of cases with a deviation from the ideal Ising behavior. Unfortunately, there is not a single parameter set of *a* and *b* in eqn (15) for the whole temperature range, since these parameters themselves depend on *T*. Consequently, the fitting of the parameters in eqn (15) has to be performed for each individual temperature with increases of Δ*T* for a particular temperature range.

Finally, the knowledge of the parameter set *a* and *b* for a particular temperature range of interest allows us to extrapolate the calculated magnetic susceptibility *χ*calcM(*n*)*T* for any arbitrary domain size *n* > 12 not directly accessible from currently feasible *ab initio* calculations. This can be especially useful to study size limits of magnetic domains in real compounds, *e.g.* induced by finite-size effects.

## 5 Results and discussion

### 5.1 Selected examples

**Fig. 2:**
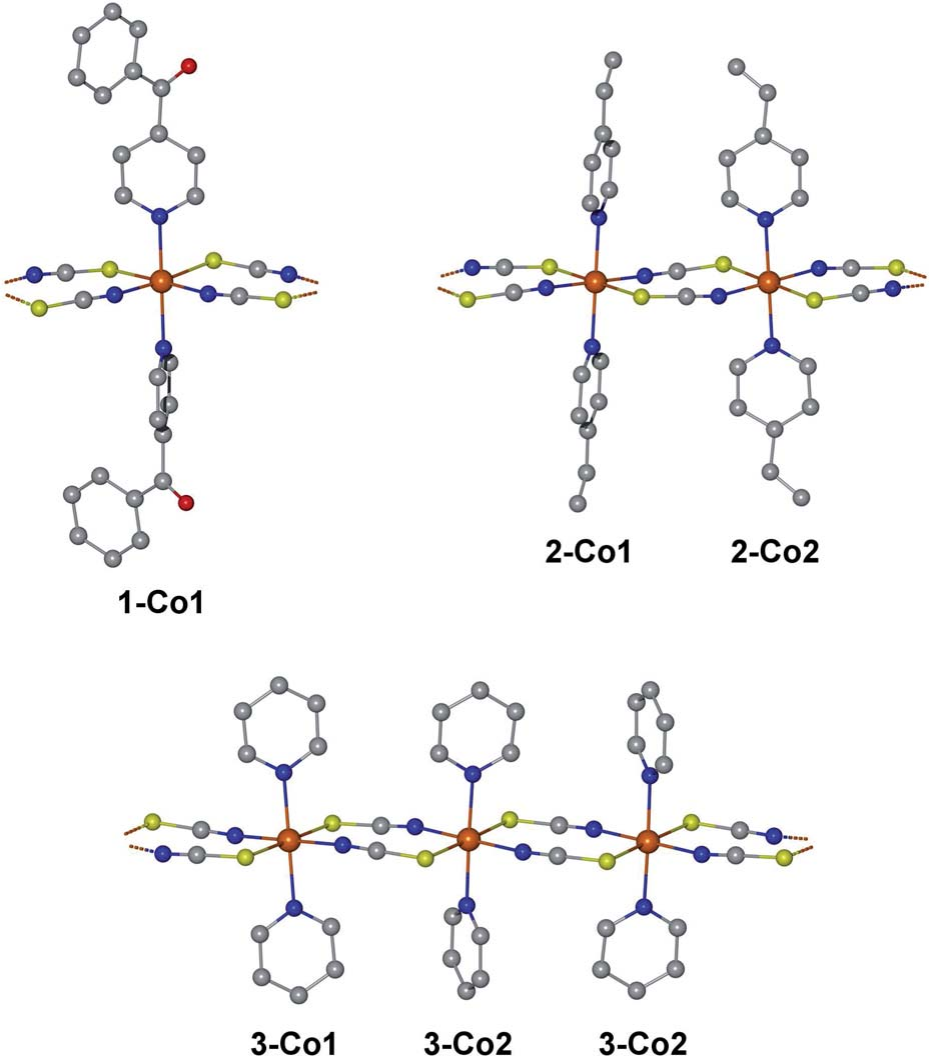
Structures of the investigated compounds **1–3** in terms of their repeating sequences. The labels below the centers name the individual fragments for the *ab initio* calculations differentiating the crystallographically independent cobalt(ii) coordination sites. Hydrogen atoms have been omitted for clarity.

We have selected three previously published cobalt(ii)-based SCMs as test cases for the herein presented approach: [Co(NCS)_2_(4-benzoylpyridine)_2_]_*n*_ (**1**), [Co(NCS)_2_(4-vinylpyridine)_2_]_*n*_ (**2**), and [Co(NCS)_2_(py)_2_]_*n*_ (**3**).^18^,^23^,^24^ The repeating sequences of the corresponding structures are depicted in Fig. 2. All three SCMs are based on cobalt(ii) ions with an [N_4_S_2_] pseudooctahedral coordination sphere, with the individual spin centers linked by two thiocyanate bridges, which mediate the ferromagnetic coupling. In addition, the coordination sphere is completed by two apical pyridine-based co-ligands (**1**: 4-benzoylpyridine; **2**: 4-vinylpyridine; **3**: pyridine).

An essential advantage of the approach presented here is that it takes into account the crystallographically independent spin centers. This allows the study of the influence of individual mononuclear fragments on the resulting properties of the magnetic domain. On this basis, it was possible to deliberately select compounds for which a different number of crystallographically independent spin centers and different repeating sequences are present within the 1D periodic chain. Compound **1** contains one crystallographically independent cobalt(ii) center (denoted as **1-Co1**) resulting in a periodic structure with a [···**1-Co1**···]_*n*_ repeating sequence. Whereas, **2** possesses two independent spin centers (**2-Co1** and **2-Co2**) leading to an alternating [···**2-Co1**···**2-Co2**···]_*n*_ repeating sequence. The main difference between both centers in **2** can be found in the orientation of the π-planes of the two 4-vinylpyridine co-ligands with respect to the chain direction (**2-Co1** perpendicular/perpendicular; **2-Co2**: parallel/parallel; see Fig. 2). Although complex **3** also contains two crystallographically independent cobalt(ii) centers (**3-Co1** and **3-Co2**), the crystal symmetry leads to an expanded repeating sequence of [···**3-Co1**···**3-Co2**···**3-Co2**···]_*n*_. Similar to **2**, the two centers in **3** show a difference in the orientation of their pyridine co-ligands (**3-Co1**: parallel/parallel; **3-Co2**: parallel/perpendicular).

In the following, the single-ion properties calculated for compounds **1–3** will be presented. Subsequently, their magnetic properties on the basis of the above described methodology utilizing the coupling scheme of an ***n*-membered spin ring** will be investigated and discussed.

### 5.2 Single-ion properties

Selected results of the single-ion properties for **1–3** obtained from *ab initio* calculations are listed in Table 1 (*cf.* Fig. S6† and Tables S2–S5†). For all spin centers in **1–3** a significant energy separation between the first and second KD is apparent, with the smallest energy separation of 139 cm^–1^ (200 K) observed for **2-Co1**. At lower temperatures, it can therefore be assumed that only the ground state KD is significantly populated in each of the paramagnetic centers. This justifies the utilization of an *S* = 1/2 effective spin Hamiltonian model, which only takes into account the well-isolated ground state KD. The large energy separation between the first and second KDs (see Table 1) is based on the pseudooctahedral coordination sphere of the cobalt(ii) ions in **1–3**, which leads to a significant splitting of the ^4^T_1g_ ground multiplet (see Tables S2 and S3†) and a high magnetic single-ion anisotropy.

**Table 1:** Components of the **g** tensor of the first two Kramers doublets (*S*_eff_ = 1/2) for the mononuclear cobalt(ii) structural models of compounds **1–3** from *ab initio* calculations

		**1-Co1**	**2-Co1**	**2-Co2**	**3-Co1**	**3-Co2**
KD1	*E*_KD1_ (cm^–1^)	0	0	0	0	0
	*g*_*x*_	1.996	1.861	2.020	2.097	1.977
	*g*_*y*_	2.251	2.979	3.761	4.231	2.466
	*g*_*z*_	7.935	7.070	6.560	6.367	7.866
KD2	*E*_KD2_ (cm^–1^)	257	139	153	182	243
	*g*_*x*_	3.099	1.669	1.157	0.863	1.661
	*g*_*y*_	2.935	1.918	1.351	1.093	2.665
	*g*_*z*_	0.764	5.621	5.638	5.438	4.166

As far as the potential validity of the Ising model is concerned, the most important property is the single-ion anisotropy, which is represented by the **g** tensor of the ground state KD. It is interesting to note that the corresponding *g*_*z*_ values show a rather large variation within the range of 6.367–7.935 (see Table 1). The corresponding easy-axis of magnetization for the investigated single-ion systems is mainly determined by the two apical pyridine-based co-ligands (angle between the N–N vector and *g*_*z*_ axis: 1.8^°^ (**1-Co1**); 0.4^°^ (**2-Co1**); 2.7^°^ (**2-Co2**); 3.3^°^ (**3-Co1**); 1.6^°^ (**3-Co2**); see Fig. S7†). The orientation of the relevant hard-axes of magnetization for the ground state KD of the individual centers is depicted in Fig. S8.† Interestingly, the *g*_*z*_ values are significantly affected by the difference in the orientation of the two pyridine π-planes in compounds **2** and **3**. The parallel orientation of the co-ligands with respect to the chain direction in **2-Co2** and **3-Co1** appears to lower the anisotropy. In fact, the largest *g*_*z*_ values of 7.935 and 7.866 are observed for **1-Co1** and **3-Co2**, both of which contain a co-ligand oriented parallel and perpendicular to the chain direction. As a result, a trend is observed for the *g*_*z*_ values depending on the orientation of the two co-ligands (see Fig. S9†): parallel/parallel < perpendicular/perpendicular < parallel/perpendicular.

### 5.3 Determination of magnetic exchange (*J*_*ij*_)

As outlined in Section 2 (see Scheme 1), for a consistent theoretical treatment, magnetic exchange which is present within a SCM must be determined from the experimental data by appropriate fitting, as described in Scheme 4. For this procedure, the lower limit of the temperature range appropriate for fitting needs to be determined, which is easily accessible from the experimental data obtained using eqn (7). In the case of compounds **1–3** this can be estimated from the experimental coupling constants *J*/*k*_B_ (**1**: 32(2), **2**: 27(3), and **3**: 28(2) K)^23^,^24^ which leads to *T*_min_ ≈ 10 K. On this basis, consistent for all three compounds, a temperature range of 10 K ≤ *T* ≤ 50 K was chosen for the fitting of the susceptibility data.

Based on the repeating sequence of the cobalt centers in compounds **1** and **2**, both can be described by a single magnetic exchange parameter. Although the repeating sequence present in compound **3** would strictly require two formally different coupling constants *J*_12_ and *J*_22_, these are assumed to be identical (*J*_12_ ≡ *J*_22_). In all cases, the *ab initio* simulations with POLY_ANISO are based on a single coupling constant and the correct repeating sequence of the crystallographically independent cobalt(ii) centers present (**1**: [···**1-Co1**···]_*n*_, **2**: [···**2-Co1**···**2-Co2**···]_*n*_, and **3**: [···**3-Co1**···**3-Co2**···**3-Co2**···]_*n*_).

The corresponding data of the experimental and fitted magnetic susceptibility for **1–3** are shown in Fig. S10–S12† as *χ*_M_*T* plots. In all cases, the calculated data (*χ*calcM*T*) well reproduce the experimental data (*χ*expM*T*) within the specified fitted range. Interestingly, similar theoretical coupling constants *J*_*ij*_ were obtained for all three compounds (**1**: *J*_11_/*k*_B_ = 4.17 K; **2**: *J*_12_/*k*_B_ = 4.89 K; **3**: *J*_12_/*k*_B_ ≡ *J*_22_/*k*_B_ = 4.82 K). The similarity observed for the magnetic exchange parameter can be explained by the presence of the same structural motif in compounds **1–3**, namely the two thiocyanate bridges, which mediates the ferromagnetic exchange. This in turn also validates the assumption of the equivalence of the two formally present parameters in compound **3**. In any case, it should be pointed out once again that these theoretical coupling constants *J*_*ij*_ cannot directly be compared with the experimentally determined ones (*J*_*ij*_ ≠ *J*), since both are based on different spin representations (see Section 4.2).

### 5.4 Energy spectrum of spin states

**Fig. 3:**
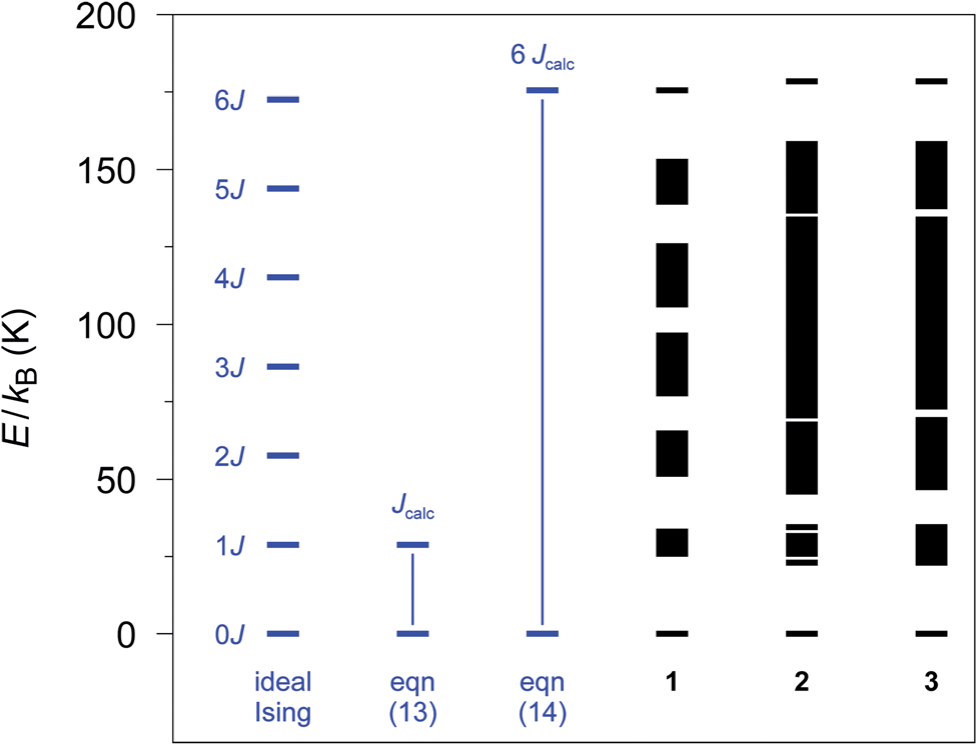
Spin states as obtained with the POLY_ANISO program employing the *ab initio* fragment calculations of **1–3**. A 12-membered spin ring coupling scheme together with the fitted coupling constants *J*_*ij*_ (see text) was used. The energy differences (blue part) are defined as in eqn (13) and (14), respectively, to obtain *J*_calc_ (*S*_eff_ = 1/2).

The relative spin state energies for the 12-membered spin rings of **1–3** calculated on the basis of the single-ion fragment properties and the fitted coupling constants *J*_*ij*_ are depicted in Fig. 3. Notably, in contrast to the Ising model, the spin states related to individual spin multiplets derived from the *ab initio* quantum mechanical simulations of **1–3** are no longer degenerate with the exception of the ground state doublet and the highest multiplet. Nevertheless, in the case of **1** all individual spin multiplets can still be distinguished, with the energies of the first excited spin multiplet found in the range of 25.4–32.5 K. For **2** and **3** a considerably wider range of energies for the first excited spin multiplet is observed (**2**: 22.7–34.4 K; **3**: 23.5–35.1 K). This effect is even more pronounced for the higher spin multiplets in **2** and **3** eventually resulting in an overlap of the energy bands of the relevant spin state multiplets.

From the energy spectra the theoretical coupling constants *J*_calc_ can be obtained from eqn (13) and (14) and are listed in Table S6† (*cf.*Fig. 3). To be independent of the apparent variation in the energy bandwidth of the first excited spin multiplet, we prefer to use the definition according to eqn (14), which leads to calculated coupling constants *J*_calc_/*k*_B_ of 29.2, 29.6, and 29.8 K for compounds **1–3**, respectively. These values are based on the same physical model as the experimental ones (*S*_eff_ = 1/2; assuming ideal Ising-behavior) and are in good agreement with the experiment (**1**: 32(2), **2**: 27(3), and **3**: 28(2) K).^23^,^24^

However, the lifting of the degeneracy for the first excited spin multiplet clearly indicates a deviation from the ideal Ising behavior of compounds **1–3** (*cf.* Fig. S2†). To further investigate this point, we have simulated the relative energy spectra for ***n*-membered spin rings** as a function of the anisotropy of the relevant spins (see Fig. S13†). It is obvious, that a lower anisotropy leads to a larger energy range for spin states belonging to the first excited multiplet. This eventually leads to a smaller energy gap between the ground state doublet and the lowest state of the first excited spin multiplet. Hence, a fully isotropic Heisenberg spin system results in a quasi-continuum of states for which the ground state doublet is no longer energetically separated. This again demonstrates the importance of a large magnetic anisotropy of the metal ions (such as cobalt(ii) ions) for the general design of SCMs.

The compounds **2** and **3** are interesting cases, since in both structures two crystallographically independent cobalt(ii) ions are present in different repeating sequences. To address the effect of such structural variations, we have simulated four hypothetical 12-membered spin rings based on mononuclear homosequences obtained from the individual cobalt centers present in **2** and **3**, assuming that the previously fitted coupling constants *J*_*ij*_ are being operative ([···M···]_*n*_ with M = **2-Co1**, **2-Co2**, **3-Co1**, and **3-Co1**). The resulting energy spectra of the spin states are depicted in Fig. S14† and clearly differ from previous results obtained for **2** and **3** (*cf.*Fig. 3). Clearly, two sets of mononuclear homosequential chains emerge from these data: (i) in the case of **2-Co1** and **3-Co2** the energy bands for the different spin multiplets are still separated, while (ii) for **2-Co2** and **3-Co1** virtually all energy bands overlap. In fact, the former two cases show a higher anisotropy and consequently a smaller deviation from the ideal Ising behavior. This agrees well with the *g*_*z*_ values of the individual centers (see Table 1). Consequently, for the cases with a higher anisotropy, this leads to a larger theoretical coupling constant *J*_calc_/*k*_B_ (**2-Co1**: 32.1 and **3-Co2**: 32.9 K; see Table S6†) as compared to the values derived for the actually present repeating sequences (**2**: 29.6 and **3**: 29.8 K). The homosequences based on the two metal centers **2-Co2** and **3-Co1** with lower single-ion anisotropy consistently lead to smaller *J*_calc_/*k*_B_ values (**2-Co2**: 28.4 and **3-Co1**: 25.9 K). This clearly shows that the resulting coupling constant *J*_calc_ is being affected by the individual single-ion anisotropies, although in all simulations, previously fitted coupling constants *J*_*ij*_ were used, which are all very similar. As a result, the lower single-ion anisotropy of the centers **2-Co2** and **3-Co1** leads to a decrease of the observed coupling constants *J*/*k*_B_ in the real compounds **2** and **3**, respectively. In summary, this clearly shows that the effect of reduced magnetic anisotropy at the metal centers constituting the SCM is within the Ising model solely hidden in the corresponding exchange parameters.

### 5.5 Anisotropy of the ground state doublet (spin ring approach)

The approach to describe the magnetic susceptibility of SCMs with the approximation of an ***n*-membered spin ring** allows us to determine the magnetic anisotropy of the ground state doublet. For compounds **1–3**, the relevant *g*_∥_ values calculated for the ground state doublet based on the coupling schemes of a 12-membered spin ring are 7.934, 6.772, and 7.321, respectively (*cf.* Table S7†). Taking into account the repeating sequence of the compounds, these values closely correspond to the average of the *g*_z_ values of the cobalt(ii) centers involved (*cf.*Table 1; **1**: *g*_*z*_****^1-Co1^**** = 7.935; **2**: (*g***2-Co1**z + *g***2-Co2***z*)/2 = 6.815; **3**: (*g***3-Co1**z + 2*g***3-Co2***z*)/3 = 7.366). However, a slight deviation from the experimental *g*_∥_ values is evident (magnetic susceptibility/HF-ESR: 7.0(2)/– (**1**); 7.3(2)/– (**2**); **3**: 7.2/7.0)^23^,^24^ and could be based on the computational models used (see Computational details).

### 5.6 Magnetic susceptibility for arbitrary domain lengths *n*

The previously fitted coupling constants *J*_*ij*_ can be used for simulations of *χ*calcM(*n*)*T* as a function of *n* (3 ≤ *n* ≤ 12). For compounds **1–3**, these simulations have been performed considering their individual repeating sequences (*cf.* Table S8† for the list of employed spin rings). Whereas for **2** and **3**, only specific values of *n* could be studied in the simulations, since the corresponding repeating sequences consist of two and three independent centers, respectively (**2**: 4, 6, 8, 10, and 12; **3**: 3, 6, 9, and 12).

**Fig. 4:**
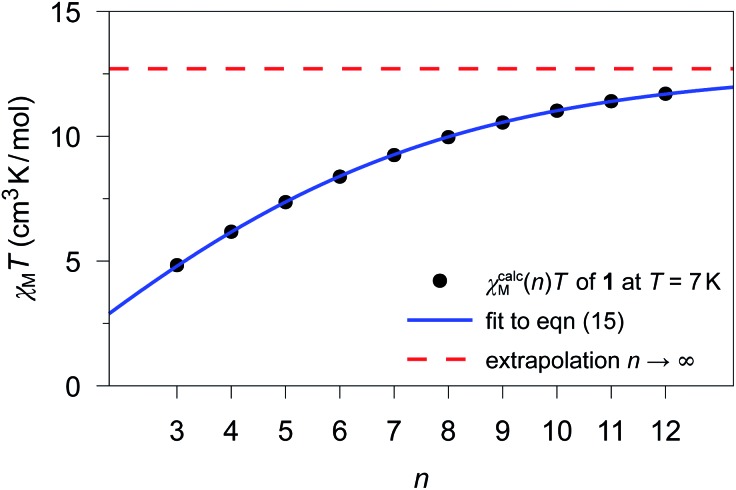
*χ* calc M (*n*)*T* values calculated for **1** at *T* = 7 K for different model sizes *n* of an ***n*-membered spin ring** employing the theoretical coupling constant *J*_11_/*k*_B_ = 4.17 K. The blue solid line represents the best fit (*a* = 12.70(2) cm^3^ K mol^–1^; *b* = 0.7673(7)) according to eqn (15) and the red dashed line represents the limit for a periodic system (*n* → ∞), which corresponds to the parameter *a*.

The fitting of *χ*calcM(*n*)*T* for **1–3** was performed according to eqn (15) as a function of *n* for all temperatures within the range 4.5 K ≤ *T* ≤ 50 K in 0.1 K increases. As an example, Fig. 4 shows the fitting of *χ*calcM(*n*)*T* for **1** at *T* = 7 K together with the extrapolated limit for *n* → ∞ (see Fig. S15 and S16† for the corresponding fits of **2** and **3**, respectively). In addition, the *ab initio*-based extrapolated *χ*calcM(*n* → ∞)*T* values for 1D periodic chains **1–3** are depicted in Fig. S17–S19.†

### 5.7 Size of magnetic domains

The extrapolated *χ*calcM(*n* → ∞)*T* values for compounds **1–3** show a deviation from the experimental data (see Fig. S17†–S19†) in the low temperature range (4.5 K ≤ *T* ≤ 10 K). For compounds **1** and **2**, the extrapolated *χ*calcM(*n* → ∞)*T* values slightly overestimate the experimental data, while in the case of **3**, *χ*calcM(*n* → ∞)*T* somewhat underestimates the experimental data. Interestingly, these deviations seem to correspond to experimental differences observed for the magnetic interchain exchange, which is antiferromagnetic in the cases of **1** and **2** (experimental *zJ*′/*k*_B_ values: –0.24(2) K (**1**) and –0.27(2) K (**2**)) and ferromagnetic for compound **3** (experimental *zJ*′/*k*_B_ = 0.5 K).^23^,^24^ This is consistent with the magnetic interchain exchange for **1–3** becoming more important at temperatures below 10 K, which in turn significantly affects the size of the magnetic domains.

**Fig. 5:**
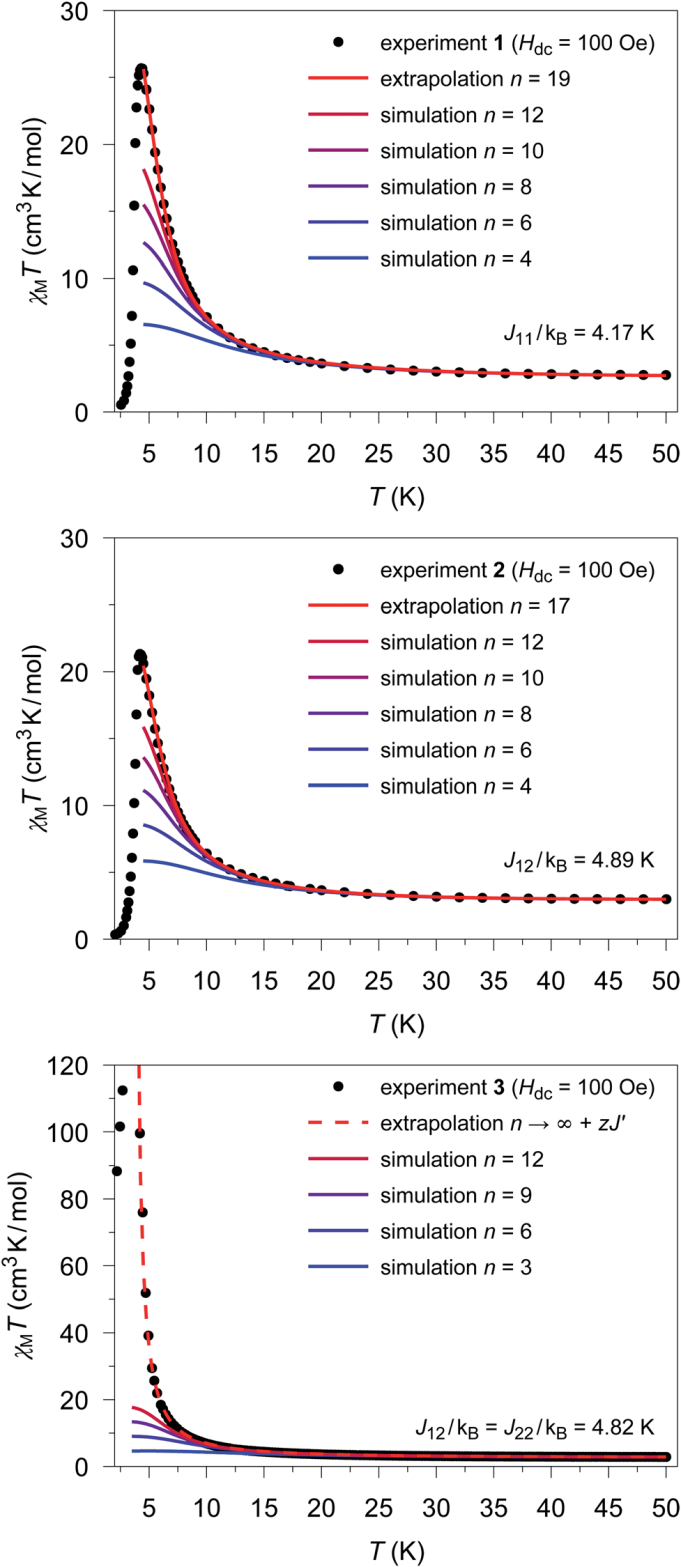
Temperature dependence of the experimental *χ*expM*T* values ([black circle]) for **1** (top), **2** (center), and **3** (bottom). Colored lines represent *χ*calcM(*n*)*T* values derived from *ab initio* fragment calculations by applying the coupling scheme of an ***n*-membered spin ring** for different values of *n* using the fitted coupling constant *J*_*ij*_ related to the Lines model. In the case of **1** and **2** the extrapolation (red solid line; for details see text) gives the best agreement with the experimental data for *n* = 19 and *n* = 17, respectively. For **3**, the dashed red line shows the mean-field corrected extrapolated magnetic susceptibility according to eqn (16) to include the effect of the ferromagnetic interchain interactions (*zJ*′/*k*_B_ = 0.85 K).

To further investigate this point, we first address the effect of *χ*calcM(*n* → ∞)*T* overestimating the experimental data, which is observed for compounds **1** and **2**. For simulations carried out for various potential sizes of the magnetic domains *n*, the best least-squares fits were obtained with *n* = 19 and *n* = 17 for **1** and **2**, respectively (see Fig. 5; fit range: 4.5 K ≤ *T* ≤ 50 K). Interestingly, the smaller domain size estimated for **2** corresponds to a somewhat stronger antiferromagnetic interchain coupling. With the given Co···Co distances (**1**: 565 pm; **2**: 559 pm) the length of the magnetic domains can be estimated to be 10.7 and 9.5 nm for compounds **1** and **2**, respectively. As indicated by the strong decrease of the experimental *χ*expM*T* values, the magnetic domains stop to grow below a temperature of about 4.5 K. This can be attributed to the antiferromagnetic interchain interactions, which become more and more dominant for temperatures below 4.5 K in both compounds. However, also finite-size effects of the real material can become relevant in this temperature range.

The situation is different for **3**, since this compound shows a ferromagnetic interchain interaction in the experiment. This is why the extrapolation of the *χ*calcM*T* values for *n* → ∞, which marks the limit of a single chain, underestimates the experimental data (*cf.* Fig. S19†). At this point, the interchain interaction cannot be neglected, and the description of compound **3** based solely on a 1D periodic Ising model becomes insufficient. However, to deal with this issue, the extrapolated *χ*calcM(*n* → ∞)*T* can be corrected using a mean-field approach according to eqn (16), with the best fit (*zJ*′/*k*_B_ = 0.85 K) depicted in Fig. 5.16
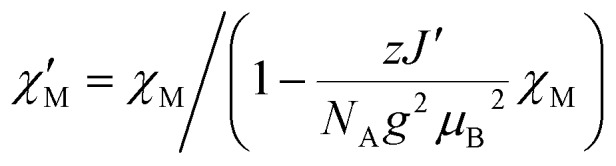



Unfortunately, the applied mean-field approach does not correct the correlation length and its temperature dependence, which prevents the determination of domains sizes within this approach. However, the domain size in **3** can be roughly estimated on the basis of eqn (4) in combination with the theoretical coupling *J*_calc_/*k*_B_ = 29.8 K derived from eqn (14) (*cf.* Section 5.4). This leads to a value of 2*ξ* ≈ 71 at a temperature of about 3.5 K, for which the experimental *χ*expM*T* shows the maximum value. This corresponds to a correlation length of about 40.0 nm for the magnetic domains, with a given Co···Co distance of 564 pm.^18^ However, the actual correlation length at this temperature can be assumed to be larger, due to the ferromagnetic interchain interactions.

## 6 Computational details

### 6.1 Structural models

*Ab initio* calculations have been performed on mononuclear cobalt(ii) fragments of the type [CoZn_2_(NCS)_4_(L)_2_]^2+^ as models for all crystallographically independent centers in **1–3** (see Fig. S6†). The computational models are based on atomic positions as obtained from the single-crystal structures of **1–3**. The positions of hydrogen atoms have been optimized at the RI-DFT^71^–^74^/BP86 75,76/def2-SVP^77^ level of theory with the Turbomole 7.1 package of programs.^78^ Within these optimizations, all cobalt(ii) ions have been replaced by zinc(ii) to save computational time in the self-consistent field steps.

### 6.2 *Ab initio* calculations

The *ab initio* CASSCF/CASPT2/RASSI-SO calculations based on the mononuclear structural models have been performed with Molcas 8.0 SP1.^79^–^81^ Relativistic effects were treated with a second-order Douglas–Kroll–Hess Hamiltonian in combination with ANO-RCC basis sets (see Table S9† for basis set information).^82^–^84^ CASSCF calculations with an active space of 7 electrons in 10 orbitals (3d and 4d shell) were carried out including 10 quartet (^4^F, ^4^P) and 40 doublet states (^2^G, ^2^P, ^2^H, ^2^D, ^2^D, ^2^F). CASPT2 calculations based on the CASSCF wave functions were subsequently performed for all quartets and the lowest 9 doublet states to adequately treat dynamic correlation effects. The RASSI-SO method was employed based on the CASSCF/CASPT2 wave functions to take spin–orbit coupling into account and allow mixing of different multiplicities. The Cartesian components of the **g** tensor, orientation of magnetic axes, and single-ion anisotropies for the mononuclear fragments were obtained with the SINGLE_ANISO module. The simulation of polynuclear magnetic properties was performed with the POLY_ANISO program employing the coupling scheme of an ***n*-membered spin ring**.^65^–^67^ Magnetic levels for the ideal Ising-type and Heisenberg-type spin systems have been obtained with the PHI v3.1.1 program.^85^

It has to be noted that the computational results presented in this work are based on mononuclear fragments of the type [CoZn_2_(NCS)_4_(L)_2_]^2+^ with zinc(ii) as terminal capping ions (see Fig. S6†). This is in contrast to the reported theoretical studies, where sodium ions have been used to exactly counterbalance the negative charges of the mononuclear cobalt(ii)-based fragments.^23^,^24^ However, the use of sodium ions leads to an overestimation of the *g*_*z*_ factors of the ground state KD (*g*_*z*_ ≥ 8). In fact, it was found that using zinc(ii) as terminal capping ions in the computational models leads to a more reliable agreement (*g*_*z*_ < 8) between the theoretical magnetic susceptibility and the experimental data and therefore has been used in this work.

### 6.3 Simulation and fitting of the magnetic susceptibility

The simulation of the magnetic susceptibility for **1–3** was performed with the POLY_ANISO program^65^–^67^ based on *ab initio* calculations for the individual structural model fragments (**1-Co1**, **2-Co1**, **2-Co2**, **3-Co1**, and **3-Co2**). Different sizes of ***n*-membered spin rings** have been calculated taking into account the correct repeating sequence and ratio of the crystallographically independent cobalt(ii) centers present in the relevant structures (*cf.* Table S8;† **1**: 3–12; **2**: 4, 6, 8, 10, and 12; **3**: 3, 6, 9, and 12). The obtained magnetic susceptibility *χ*^calc^ was divided by *n* to obtain the molar susceptibility *χ*calcM which corresponds to one cobalt(ii) ion. The calculations of the magnetic susceptibility were solely based on the ground state KD of each spin center (*S*_eff_ = 1/2) to keep the number of spin states low and the computational effort feasible.

In addition, the simulation of the magnetic susceptibility requires knowledge of the magnetic exchange between neighboring spins (*J*_*ij*_). To obtain these theoretical coupling constants, fitting has been performed for compounds **1–3** by iteratively varying the coupling constants *J*_*ij*_ so that the sum of the squared residuals between the calculated and the experimental *χ*_M_*T* values is minimized (*cf.*Scheme 4). These fitting experiments are performed on the basis of the coupling scheme of a 12-membered spin ring for a temperatures range specific for the individual compound (**1–3**: 10 K ≤ *T* ≤ 50 K), where the lower limit of this temperature range (*T*_min_) can be determined from eqn (7).

It should to be noted, however, at this point that generally an offset is observed between the absolute experimental and simulated *χ*_M_*T* values. In principle, this offset can be attributed to various causes, which are based either on the approximations used in the calculation methods or on experimental uncertainties, such as the presence of slight paramagnetic impurities. In this context, it is also relevant that the simulations performed with POLY_ANISO solely take into account the ground state KD of the treated single-ion centers and, therefore, neglect the contributions of higher spin–orbit states of the ^4^T_1g_ ground multiplet to the magnetic susceptibility. As an example, Fig. S20†–S22† show the calculated *χ*_M_*T* values for **1–3** as obtained by a coupling scheme of a 6-membered spin ring, however, additionally taking into account the first excited KD (accounting for 4^6^ ≡ 4096 microstates). Therefore, in order to allow for the fitting procedure required for the determination of the coupling constant *J*_*ij*_ (*cf.*Scheme 4) the calculated *χ*_M_*T* values have to be adjusted by a scaling factor, which is obtained from the ratio of the simulated and experimental susceptibility at *T* = 50 K.

## 7 Conclusions

In this work, we present an approach that allows us to directly relate the results derived from high-level *ab initio* quantum mechanical calculations on mononuclear fragments to the experimental magnetic data of 1D periodic compounds. To link theory and experiment, a spin coupling scheme is necessary which describes the interactions between the neighboring spins. In this context, we could prove that the coupling scheme of an ***n*-membered spin ring** is superior to that of an ***n*-membered open chain**, since the former model for an identical value of *n* better reproduces the correlation length of a periodic chain within a given temperature range.

In order to follow this approach, in addition to the electronic structure of the individual spin centers, the exchange coupling between them is required for a full *ab initio* simulation of the magnetic susceptibility for an ***n*-membered spin ring**. The latter, however, is not available from *ab initio* calculations for the systems in question. Instead, in order to correlate the available *ab initio* description of the individual spin centers with experimental data, the intrachain coupling as given by the Lines model (*J*_*ij*_) is determined by fitting against the experimental data. This fitting procedure was successfully applied to three selected cobalt(ii)-based SCMs, as examples containing three different repeating sequences. For the low temperature range, the given approach allows us to reproduce the experimental properties of 1D periodic compounds, such as the magnetic susceptibility, from *ab initio* calculations on single spin centers in combination with the coupling scheme of a 12-membered spin ring. Moreover, this provides the basis for the calculation of the energy spectra of spin states within the given coupling scheme. The structure of these spectra and in particular the broadening observed for the states related to the first excited spin multiplet are characteristic of the magnetic anisotropy of the individual spin centers building the 1D periodic compound. In fact, for all three investigated test cases **1–3** the observed lifting of the degeneracy of the first excited spin multiplet clearly indicates the presence of a significant deviation from a pure Ising anisotropy. From the total spread of the spectra based on the coupling scheme of a 12-membered spin ring it is possible to derive a theoretical exchange coupling constant *J*_calc_, which can be directly compared to the experimental one. To the best of our knowledge, this parameter, which is important for 1D periodic magnetic chains, has not yet been determined by theory. In addition, this approach allows for the simulation of hypothetical chains that exclusively reflect the properties of one of the independent spin centers of a chain, which may initially contain multiple independent spin centers and more complex repeating sequences. For compounds **2** and **3** this shows that the deviation from the ideal Ising anisotropy for the spin centers affects the absolute values of the coupling constant *J*_calc_, indicating the possible contribution of anisotropy effects on experimental exchange parameters.

An additional advantage of this approach is the ability to simulate spin rings of different sizes using the fitted intrachain coupling constants *J*_*ij*_, respecting the repeating sequence of the independent magnetic centers within the chains. This allows us to determine the magnetic susceptibility as a function of the ring size *n* and subsequently to extrapolate for a 1D periodic system (*n* → ∞). The extrapolation toward larger magnetic domains (*n* > 12) gives access to magnetic properties at temperatures lower than the temperature range used for fitting (*T* < *T*_min_). In fact, this extrapolation technique could be used to estimate the statistical mean length of the magnetic domains in the SCM test cases **1–3**.

The method presented herein can be beneficial for studies of other SCM systems which contain spin centers with significantly different single-ion magnetic anisotropies, *e.g.* heterometallic compounds. An appropriate modelling of magnetic domains by theory as shown in this work is a first step towards the investigation of dynamic magnetic properties in SCM compounds by theoretical methods and will be part of our future investigations. In summary, the approach presented provides a valuable contribution from quantum theory to investigate SCMs and has the potential to improve their future design.

## Conflicts of interest

There are no conflicts to declare.

## Supplementary Material

Supplementary information
