# Supplementary material for: How to link theory and experiment for single-chain magnets beyond the Ising model: magnetic properties modeled from ab initio calculations of molecular fragments
Source: Chem Sci. 2019 Aug 19;10(40):9189–202. doi: 10.1039/c9sc02735a (PMC6979495; doi:10.1039/c9sc02735a)
Supplement: Supplementary file 1 [file SC-010-C9SC02735A-s001.pdf]

Electronic Supplementary Material (ESI) for Chemical Science.
This journal is © The Royal Society of Chemistry 2019

# How to link theory and experiment for single-chain magnets beyond the Ising model: magnetic properties modeled from *ab initio* calculations of molecular fragments

Michael Böhme and Winfried Plass

**Electronic Supplementary Information (ESI)**

## Contents


1 Comparison of coupling schemes S2

2 Extrapolation of the correlation length S3

3 Correlation length and coupling schemes S4

4 Magnetic susceptibility of a 1D periodic Ising chain S6

5 *Ab initio* computational models and single-ion properties S7

6 Fit of the magnetic susceptibility S14

7 Determined magnetic coupling constants $J_{\text{calc}}$ S17

8 Spin states in dependence on the single-ion anisotropy S18

9 Additional POLY_ANISO results S19

10 Extrapolation of the magnetic susceptibility S21

11 Magnetic interchain interactions S22

12 Basis set information S25

13 Decomposition of the calculated magnetic susceptibility S26

# 1 Comparison of coupling schemes

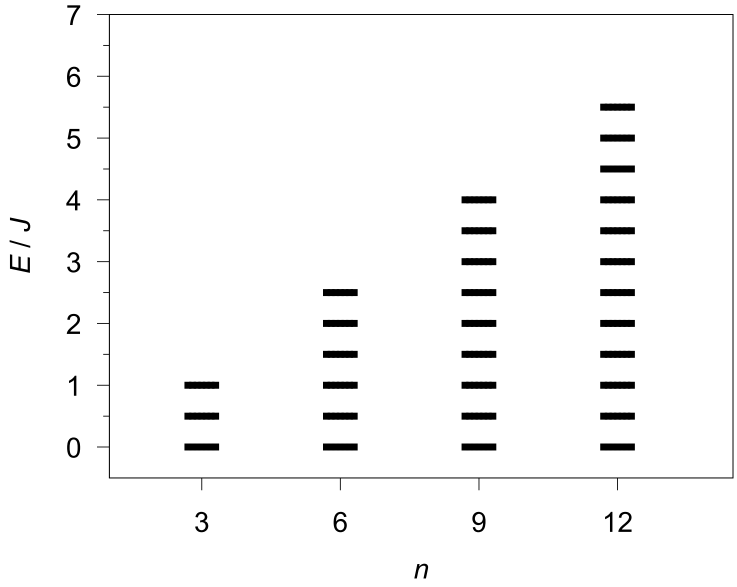


**Fig. S1:** Energy spectrum scaled in units of $J$ for an ***n*-membered open chain** as obtained from the Hamiltonian given in eqn (1) from the main manuscript for selected model sizes $n$.

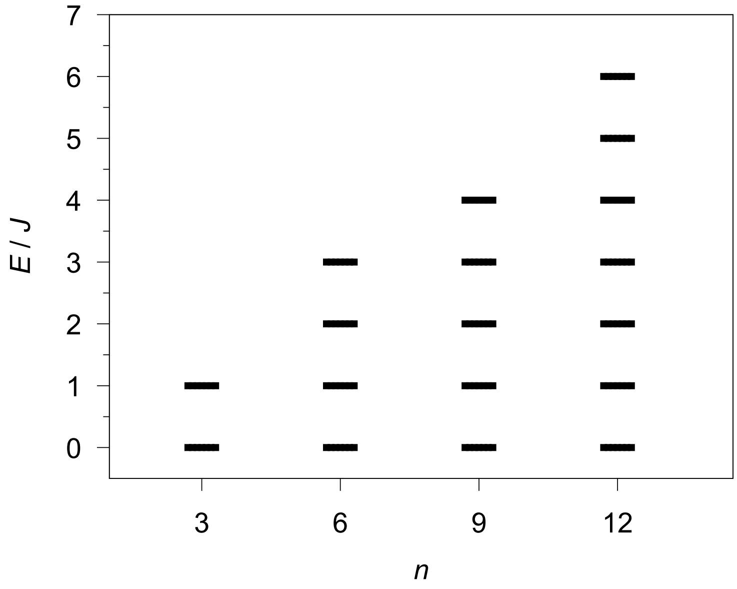


**Fig. S2:** Energy spectrum scaled in units of $J$ for an ***n*-membered spin ring** as obtained from the Hamiltonian given in eqn (2) from the main manuscript for selected model sizes $n$.

## 2 Extrapolation of the correlation length

The correlation length $2\xi$ for a 1D periodic chain of Ising-type spins ($S_{\text{eff}} = {}^{1}/_{2}$) is defined as in eqn (4) of the main manuscript. By assuming the correlation function $\gamma$ for an Ising spin as given in eqn (3), one can show that the correlation lengths $2\xi_{\text{chain}}(n)$ and $2\xi_{\text{ring}}(n)$ as given in eqns (5) and (6) of the main manuscript are identical to eqn (4) for $n \to \infty$.

$$\lim_{n\to\infty} 2\xi_{\text{chain}}(n) = \frac{1+\gamma}{1-\gamma} - \underbrace{\frac{2\gamma}{n}\frac{1-\gamma^{n}}{(1-\gamma)^{2}}}_{=0} = \exp\left(\frac{J}{2k_{\text{B}}T}\right) \tag{S1}$$

$$\lim_{n\to\infty} 2\xi_{\text{ring}}(n) = \frac{1+\gamma}{1-\gamma} \cdot \underbrace{\frac{1-\gamma^{n}}{1+\gamma^{n}}}_{=1} = \exp\left(\frac{J}{2k_{\text{B}}T}\right) \tag{S2}$$

## 3 Correlation length and coupling schemes

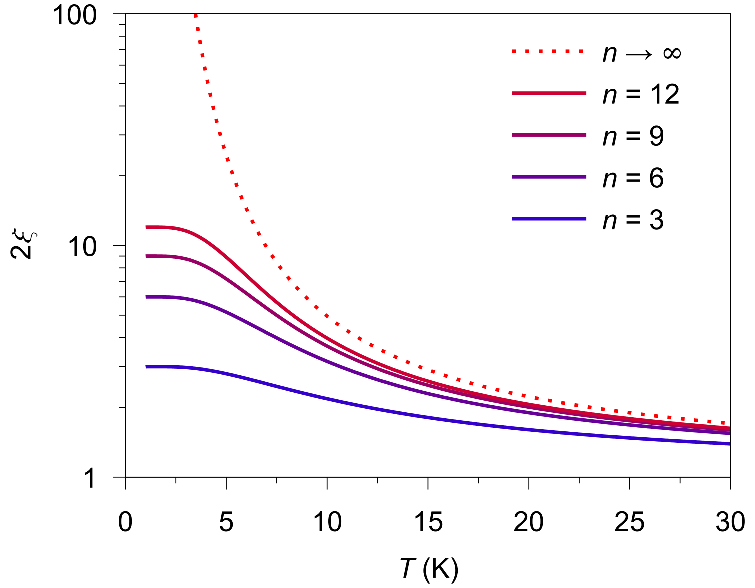


**Fig. S3:** Temperature dependence of the correlation length $2\xi_{\mathrm{chain}}$ for selected ***n*-membered open chains** of spins as obtained from eqn (5) from the main manuscript for Ising-type spins ($J/k_{\mathrm{B}} = 32\,\mathrm{K}$). The dotted line shows the extrapolation ($n \to \infty$) for a 1D periodic chain ($2\xi_{\infty}$).

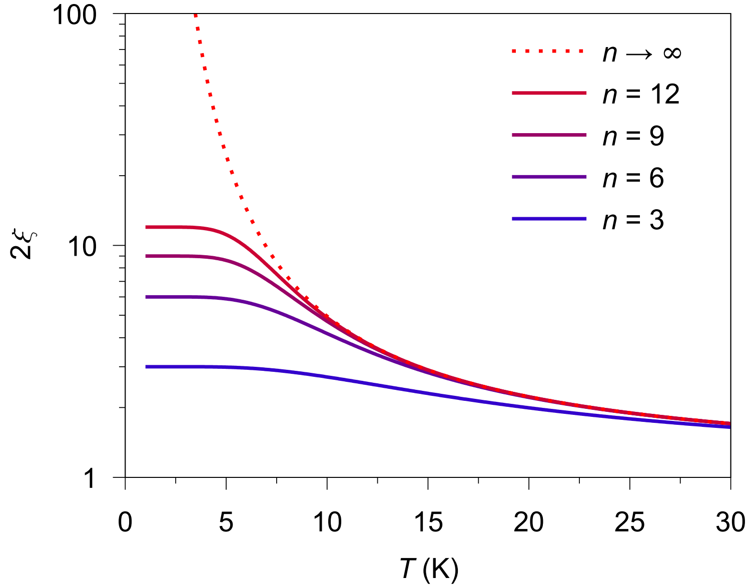


**Fig. S4:** Temperature dependence of the correlation length $2\xi_{\mathrm{ring}}$ for selected ***n*-membered spin rings** as obtained from eqn (6) from the main manuscript for Ising-type spins ($J/k_{\mathrm{B}} = 32\,\mathrm{K}$). The dotted line shows the extrapolation ($n \to \infty$) for a 1D periodic chain ($2\xi_{\infty}$).

**Table S1:** Temperature dependence of the correlation length $2\xi$ for a 1D periodic chain ($2\xi_\infty$) and the two different coupling schemes ($2\xi_{\text{chain}}(n = 12)$: 12-membered open chain; $2\xi_{\text{ring}}(n = 12)$: 12-membered spin ring) as obtained from eqns (4)–(6) from the main manuscript for Ising-type spins and assuming a ferromagnetic exchange interaction ($J/k_B$ = 32 K). $\gamma$ represents the correlation function for Ising spins as given in eqn (3) from the main manuscript.

| $T$ (in K) | $\gamma$ | $2\xi_\infty$ | $2\xi_{\text{chain}}(n = 12)$ | $2\xi_{\text{ring}}(n = 12)$ |
|---:|---:|---:|---:|---:|
| 5 | 0.92167 | 24.53 | 8.90 | 11.13 |
| 10 | 0.66404 | 4.95 | 3.98 | 4.88 |
| 15 | 0.48792 | 2.91 | 2.60 | 2.90 |
| 20 | 0.37995 | 2.23 | 2.06 | 2.23 |
| 25 | 0.30951 | 1.90 | 1.79 | 1.90 |
| 30 | 0.26052 | 1.70 | 1.63 | 1.70 |
| 35 | 0.22467 | 1.58 | 1.52 | 1.58 |
| 40 | 0.19738 | 1.49 | 1.44 | 1.49 |
| 45 | 0.17593 | 1.43 | 1.38 | 1.43 |
| 50 | 0.15865 | 1.38 | 1.34 | 1.38 |
| 55 | 0.14444 | 1.34 | 1.30 | 1.34 |
| 60 | 0.13255 | 1.31 | 1.28 | 1.31 |
| 65 | 0.12246 | 1.28 | 1.25 | 1.28 |
| 70 | 0.11379 | 1.26 | 1.23 | 1.26 |
| 75 | 0.10626 | 1.24 | 1.22 | 1.24 |
| 80 | 0.09967 | 1.22 | 1.20 | 1.22 |
| 85 | 0.09384 | 1.21 | 1.19 | 1.21 |
| 90 | 0.08866 | 1.19 | 1.18 | 1.19 |
| 95 | 0.08401 | 1.18 | 1.17 | 1.18 |
| 100 | 0.07983 | 1.17 | 1.16 | 1.17 |

## 4 Magnetic susceptibility of a 1D periodic Ising chain

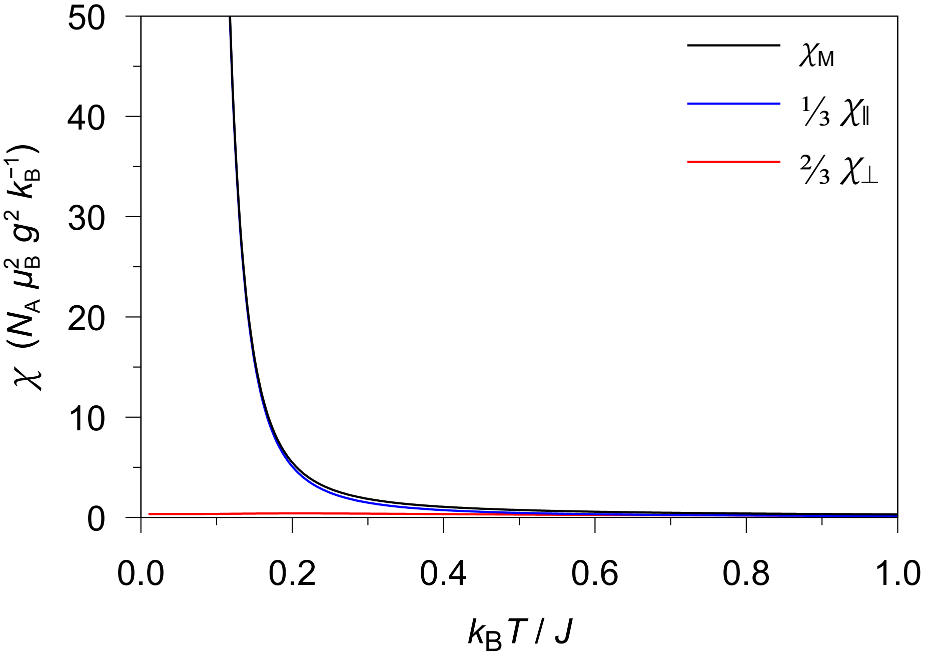


**Fig. S5:** Magnetic susceptibility $\chi$ (in $N_A \mu_B^2 g^2 k_B^{-1}$) for a 1D periodic Ising chain (black line; $S_{eff} = {}^1/_2$; $J/k_B$ = 32 K) as obtained by eqns (8)–(10) from the main manuscript. The molar magnetic susceptibility $\chi_M$ can be decomposed into two parts with respect to the orientation of the spins: parallel (${}^1/_3\ \chi_\parallel$; blue line) and perpendicular (${}^2/_3\ \chi_\perp$; red line).

## 5 *Ab initio* computational models and single-ion properties

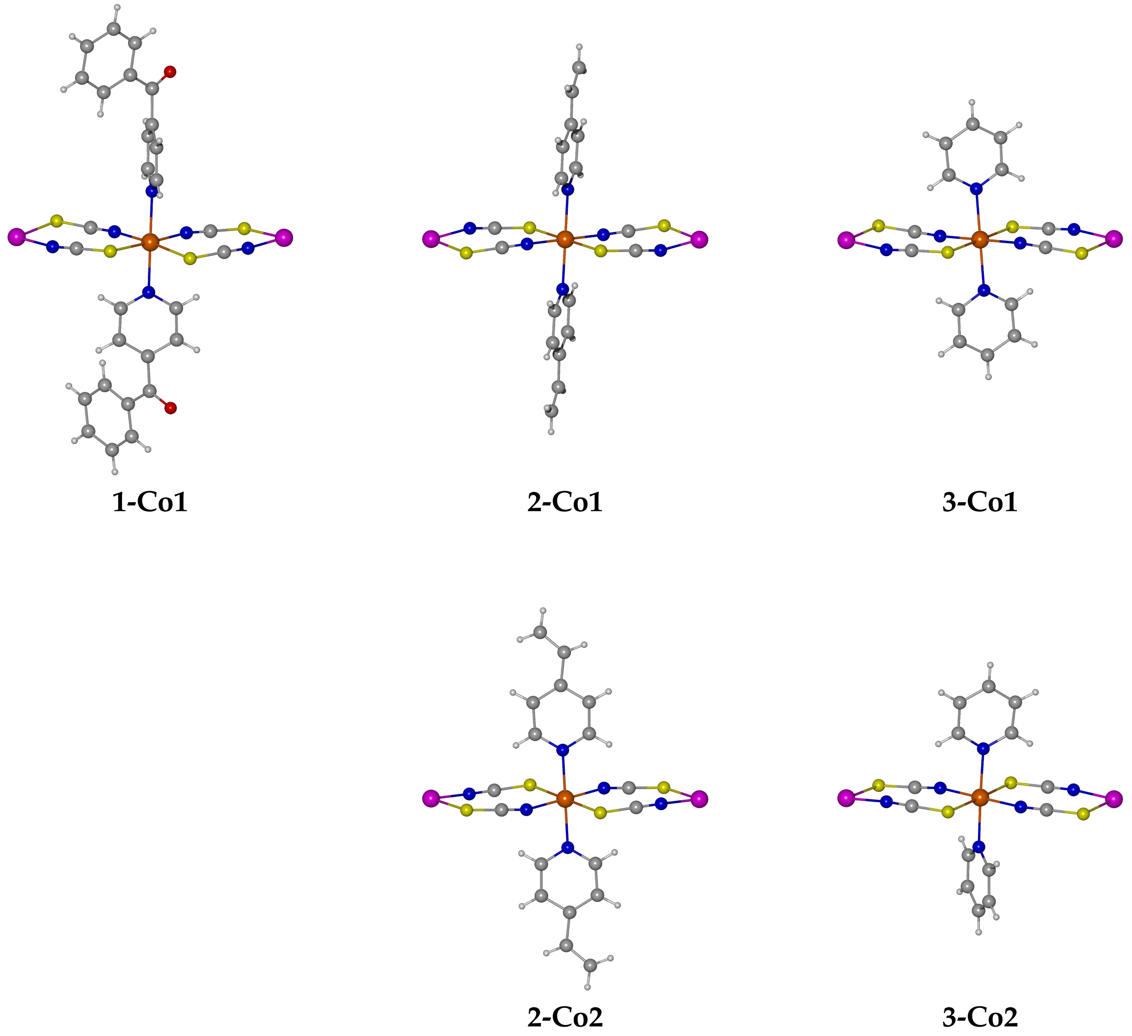


**Fig. S6:** *Ab initio* computational models for the crystallographically independent cobalt(II) centers in **1**–**3** (color code: Co – orange; Zn – pink).

**Table S2:** Relative CASSCF energies (in $cm^{-1}$) of all quartet and the nine lowest doublet states for the mononuclear cobalt(II) computational models.

| $2S+1$ | Term | Subterm | **1-Co1** | **2-Co1** | **2-Co2** | **3-Co1** | **3-Co2** |
|---|---|---|---|---|---|---|---|
| 4 | $^4F$ | $^4T_{1g}$ | 0 | 0 | 0 | 0 | 0 |
| | | | 115 | 770 | 588 | 475 | 155 |
| | | | 919 | 1265 | 1155 | 998 | 870 |
| | | $^4T_{2g}$ | 6994 | 5780 | 5590 | 5382 | 5380 |
| | | | 7157 | 8236 | 7916 | 7608 | 7600 |
| | | | 7695 | 8756 | 8936 | 8532 | 8502 |
| | | $^4A_{2g}$ | 15379 | 15882 | 15850 | 15259 | 15367 |
| | $^4P$ | $^4T_{1g}$ | 21052 | 21318 | 21488 | 21390 | 21257 |
| | | | 23702 | 23250 | 22696 | 22270 | 22423 |
| | | | 24212 | 26275 | 26529 | 26188 | 25927 |
| 2 | $^2G$ | | 12697 | 11986 | 11742 | 12024 | 11801 |
| | | | 14754 | 15987 | 16039 | 16121 | 15727 |
| | | | 19101 | 18071 | 17595 | 17728 | 17660 |
| | | | 19129 | 18519 | 18501 | 18532 | 18424 |
| | | | 19199 | 19413 | 19210 | 19147 | 19071 |
| | | | 20315 | 20101 | 20068 | 20078 | 19801 |
| | | | 20414 | 21036 | 20957 | 20808 | 20642 |
| | | | 20482 | 21495 | 21401 | 21273 | 21166 |
| | | | 24619 | 24351 | 24354 | 24299 | 24181 |

**Table S3:** Relative CASPT2 energies (in $cm^{-1}$) of all quartet and the nine lowest doublet states for the mononuclear cobalt(II) computational models.

| $2S+1$ | Term | Subterm | **1-Co1** | **2-Co1** | **2-Co2** | **3-Co1** | **3-Co2** |
|---|---|---|---|---|---|---|---|
| 4 | $^4F$ | $^4T_{1g}$ | 0 | 0 | 0 | 0 | 0 |
| | | | 114 | 807 | 647 | 506 | 241 |
| | | | 860 | 1148 | 1030 | 853 | 844 |
| | | $^4T_{2g}$ | 7629 | 6808 | 6597 | 6344 | 6509 |
| | | | 7764 | 9370 | 8982 | 8623 | 8789 |
| | | | 8971 | 9798 | 9980 | 9516 | 9659 |
| | | $^4A_{2g}$ | 17578 | 18030 | 18035 | 17355 | 17618 |
| | $^4P$ | $^4T_{1g}$ | 18522 | 18793 | 18996 | 18863 | 18867 |
| | | | 21190 | 21190 | 20564 | 20035 | 20385 |
| | | | 21738 | 24064 | 24375 | 23975 | 23820 |
| 2 | $^2G$ | | 9955 | 9276 | 9018 | 9318 | 9225 |
| | | | 12128 | 13194 | 13270 | 13373 | 13079 |
| | | | 16686 | 15909 | 15543 | 15705 | 15764 |
| | | | 16471 | 16462 | 16389 | 16403 | 16433 |
| | | | 16625 | 16789 | 16589 | 16547 | 16596 |
| | | | 17613 | 17347 | 17324 | 17361 | 17187 |
| | | | 17080 | 18341 | 18242 | 18108 | 18074 |
| | | | 17920 | 18614 | 18535 | 18459 | 18448 |
| | | | 20882 | 20771 | 20650 | 21114 | 20732 |

**Table S4:** Relative RASSI-SO energies (in $cm^{-1}$) of the $^4T_{1g}[^4F]$ ground multiplet for the mononuclear cobalt(II) computational models.

| Kramers doublet | **1-Co1** | **2-Co1** | **2-Co2** | **3-Co1** | **3-Co2** |
|---|---|---|---|---|---|
| 1 | 0 | 0 | 0 | 0 | 0 |
| 2 | 257 | 139 | 153 | 182 | 243 |
| 3 | 487 | 888 | 787 | 707 | 538 |
| 4 | 800 | 1127 | 1022 | 969 | 866 |
| 5 | 1392 | 1466 | 1391 | 1290 | 1311 |
| 6 | 1420 | 1576 | 1497 | 1391 | 1396 |

**Table S5:** Calculated zero-field splitting parameters and their Cartesian components of the $g$ tensor ($S_{eff} = 3/2$) for the mononuclear cobalt(II) computational models.

| | **1-Co1** | **2-Co1** | **2-Co2** | **3-Co1** | **3-Co2** |
|---|---|---|---|---|---|
| $D$ ($cm^{-1}$) | 114.18 | 63.86 | 73.64 | 89.15 | 109.50 |
| $E$ ($cm^{-1}$) | 33.74 | 15.59 | 24.55 | 10.65 | 30.71 |
| $E/D$ | 0.30 | 0.24 | 0.33 | 0.12 | 0.28 |
| $g_x$ | 1.722 | 2.743 | 2.745 | 2.811 | 1.801 |
| $g_y$ | 2.002 | 2.435 | 2.527 | 2.623 | 2.222 |
| $g_z$ | 3.130 | 2.000 | 1.947 | 1.869 | 3.072 |

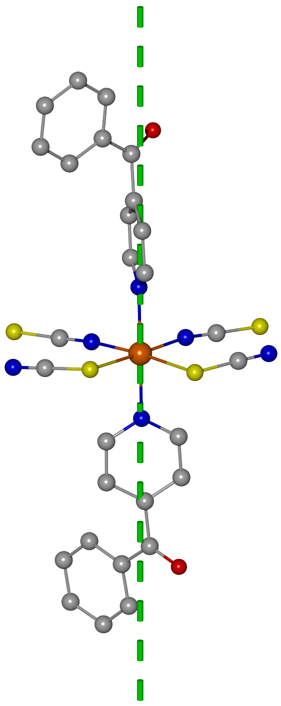

(a) **1-Co1**

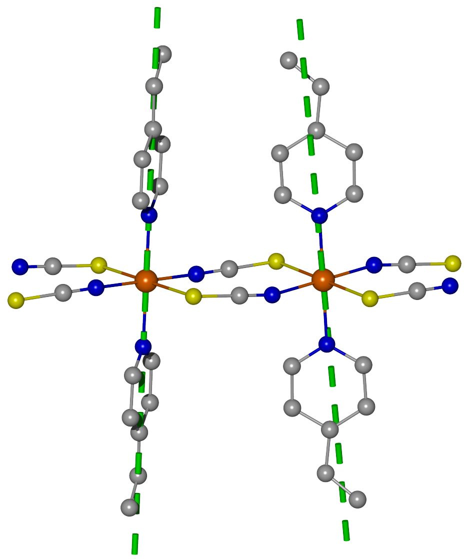

(b) **2-Co1**· · · **2-Co2**

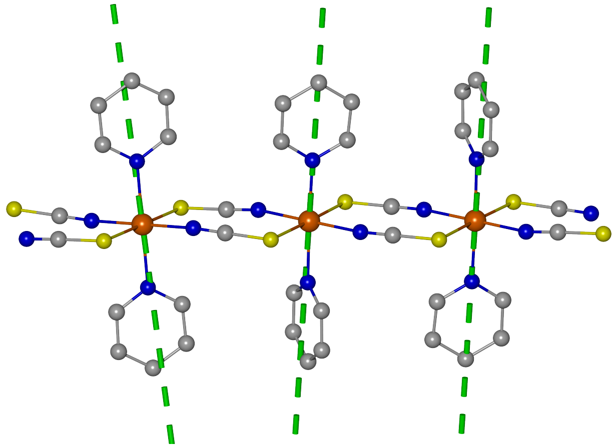

(c) **3-Co1**· · · **3-Co2**· · · **3-Co2**

**Fig. S7:** Easy-axis of magnetization from *ab initio* calculations of the ground state KD ($S_{\mathrm{eff}} = 1/2$) for the individual centers projected onto the smallest repeating sequence of the chain structures for **1**–**3**. Hydrogen atoms have been omitted for clarity.

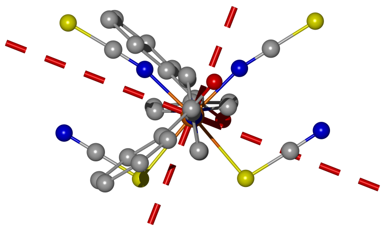

(a) **1-Co1**

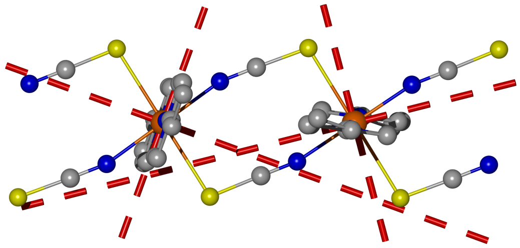

(b) **2-Co1**$\cdots$**2-Co2**

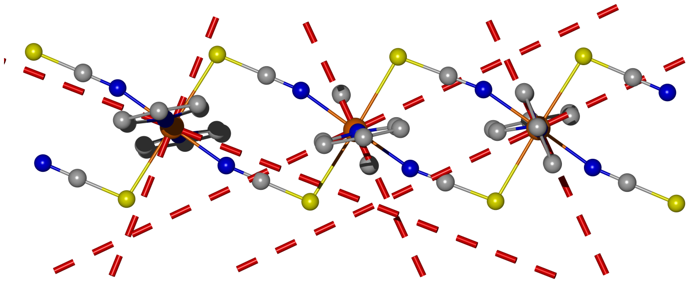

(c) **3-Co1**$\cdots$**3-Co2**$\cdots$**3-Co2**

**Fig. S8:** Hard-axes of magnetization from *ab initio* calculations of the ground state KD ($S_{\text{eff}} = 1/2$) for the individual centers projected onto the smallest repeating sequence of the chain structures for **1**–**3** from a top view. Hydrogen atoms have been omitted for clarity.

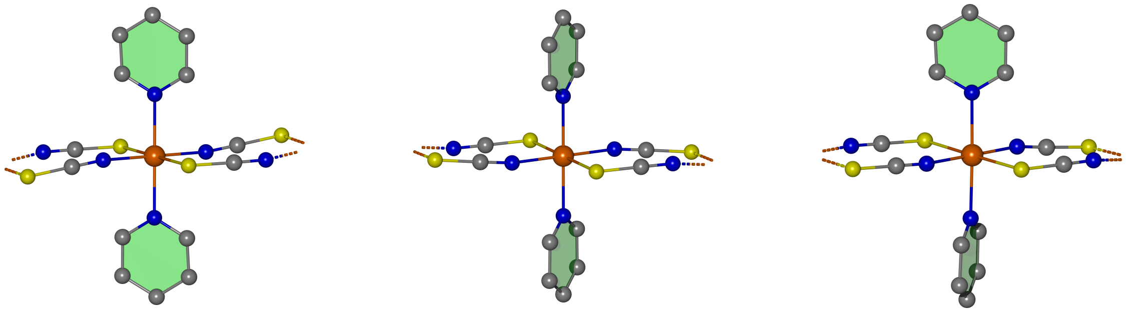

parallel/parallel | perpendicular/perpendicular | parallel/perpendicular

**2-Co2** (6.560) | **2-Co1** (7.070) | **1-Co1** (7.935)

**3-Co1** (6.367) | | **3-Co2** (7.866)

→ increasing cobalt(II) single-ion anisotropy

**Fig. S9:** Classification of the crystallographically independent cobalt(II) centers of **1**–**3** in three groups depending on the orientation of the $\pi$-planes (in green) of both pyridine-based co-ligands with respect to the direction of the thiocyanate chain (hydrogen atoms have been omitted for clarity). The values in the parentheses are the corresponding Cartesian $g_z$ values of the ground state KD.

## 6 Fit of the magnetic susceptibility

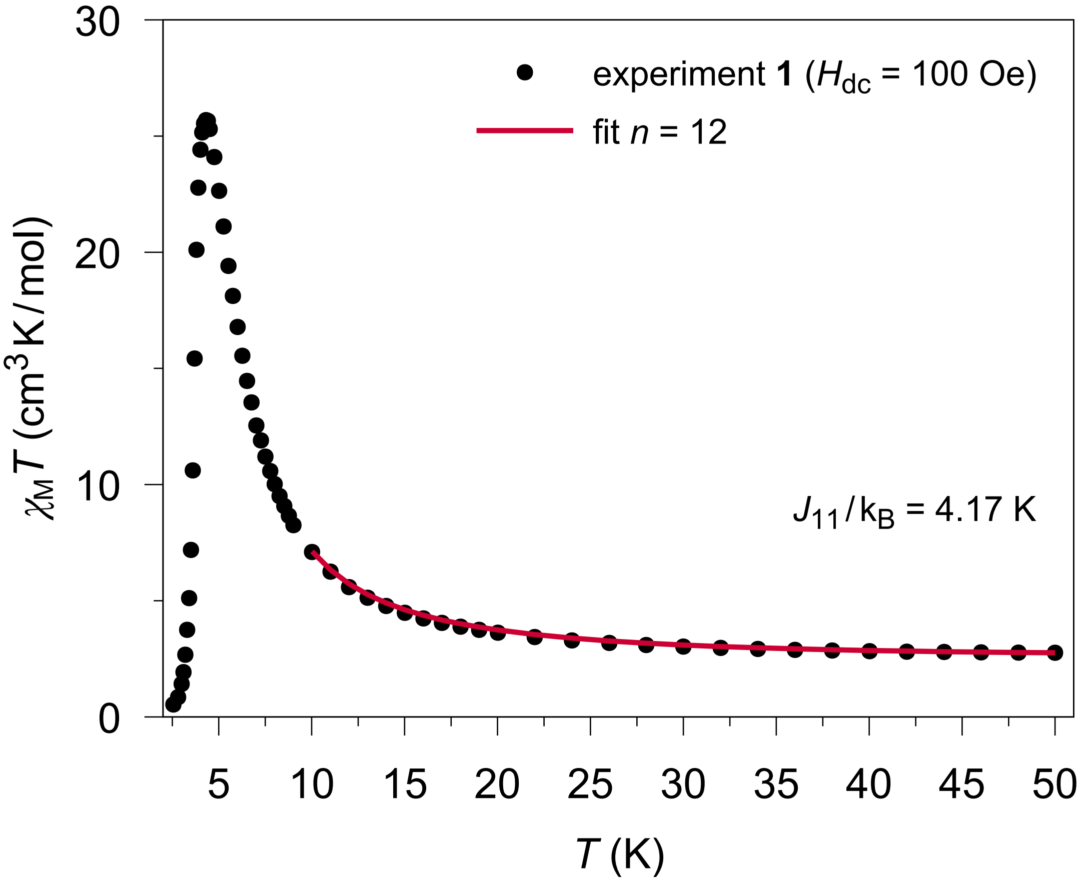


**Fig. S10:** Experimental and simulated temperature dependence of $\chi_M T$ (•) for **1**. The POLY_ANISO program on basis of the *ab initio* calculations was used to simulate the magnetic susceptibility employing a 12-membered spin ring coupling scheme in the temperature range of $10\,\mathrm{K} \leq T \leq 50\,\mathrm{K}$. The root-mean-square deviation between the experimental and theoretical magnetic susceptibility was minimized by adjusting the theoretical coupling constant $J_{11}$ between the individual centers. The red line shows the best fit of the magnetic susceptibility based on the *ab initio* calculations.

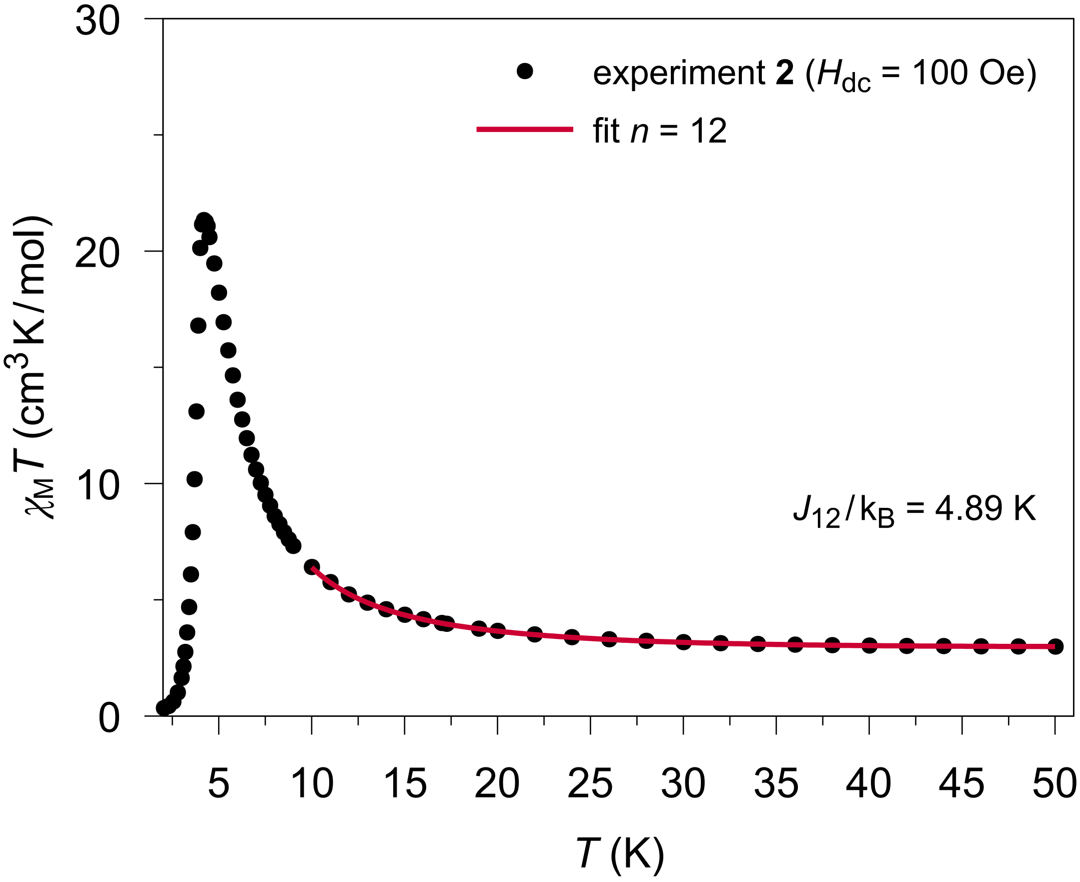


**Fig. S11:** Experimental and simulated temperature dependence of $\chi_M T$ (•) for **2**. The POLY_ANISO program on basis of the *ab initio* calculations was used to simulate the magnetic susceptibility employing a 12-membered spin ring coupling scheme in the temperature range of $10\,\mathrm{K} \leq T \leq 50\,\mathrm{K}$. The root-mean-square deviation between the experimental and theoretical magnetic susceptibility was minimized by adjusting the theoretical coupling constant $J_{12}$ between the individual centers. The red line shows the best fit of the magnetic susceptibility based on the *ab initio* calculations.

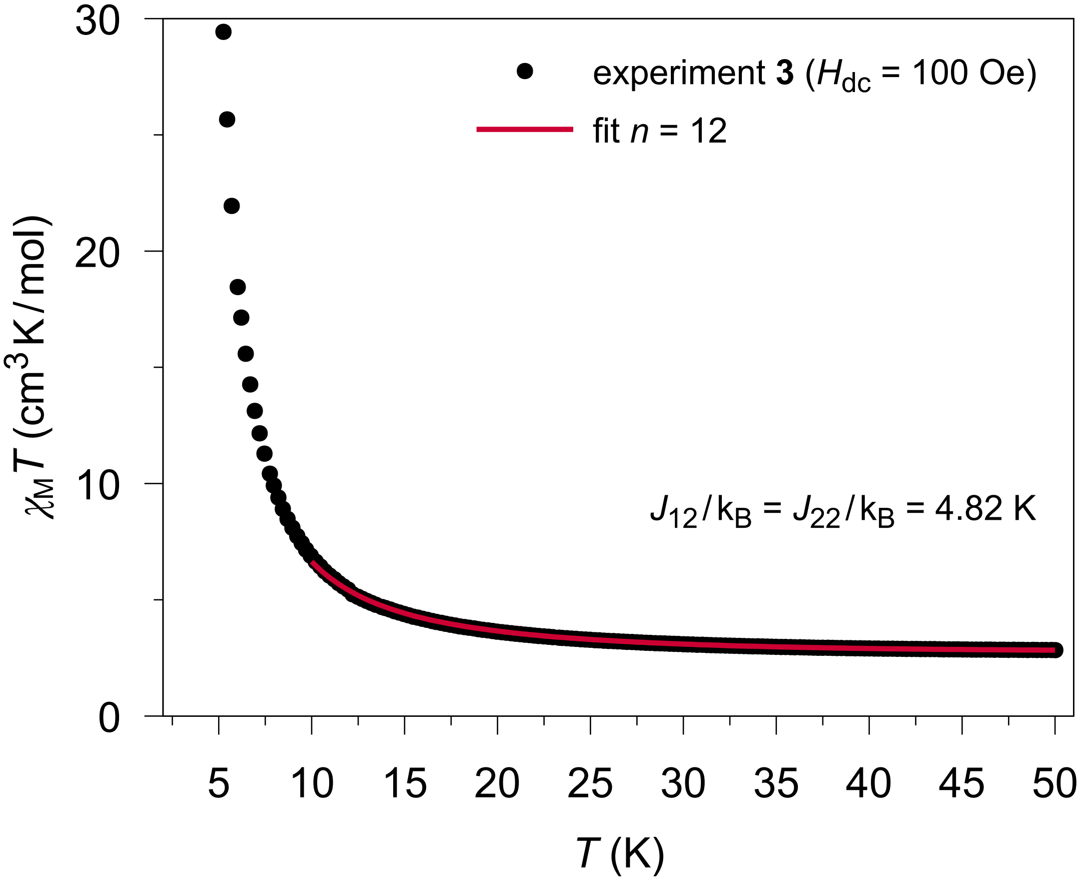


**Fig. S12:** Experimental and simulated temperature dependence of $\chi_M T$ (•) for **3**. The POLY_ANISO program on basis of the *ab initio* calculations was used to simulate the magnetic susceptibility employing a 12-membered spin ring coupling scheme in the temperature range of $10\,K \leq T \leq 50\,K$. The root-mean-square deviation between the experimental and theoretical magnetic susceptibility was minimized by adjusting the theoretical coupling constants $J_{12}$ ($\equiv J_{22}$) between the individual centers. The red line shows the best fit of the magnetic susceptibility based on the *ab initio* calculations.

# 7 Determined magnetic coupling constants $J_{\mathrm{calc}}$

**Table S6:** Coupling constants (in K) for **1**–**3** and four hypothetical chains (**2-Co1**, **2-Co2**, **3-Co1**, **3-Co2**) as obtained from the simulation of 12-membered spin rings employing the POLY_ANISO program on the base of *ab initio* fragment calculations and the fitted coupling constants of the Lines model $J_{ij}$ .

| Compound | Simulated system | $J_{\mathrm{calc}}/k_{\mathrm{B}}$ eqn (13) | $J_{\mathrm{calc}}/k_{\mathrm{B}}$ eqn (14) | $J/k_{\mathrm{B}}$ exp. |
|---|---|---|---|---|
| **1** | $[\cdots$ **1-Co1** $\cdots]_{12}$ | 28.8 | 29.2 | 32(2) |
| **2** | $[\cdots$ **2-Co1** $\cdots$ **2-Co2** $\cdots]_{6}$ | 28.5 | 29.6 | 27(3) |
| **3** | $[\cdots$ **3-Co1** $\cdots$ **3-Co2** $\cdots$ **3-Co2** $\cdots]_{4}$ | 29.0 | 29.8 | 29(2) |
| Hypothetical chains | | | | |
| **2-Co1** | $[\cdots$ **2-Co1** $\cdots]_{12}$ | 31.4 | 32.1 | – |
| **2-Co2** | $[\cdots$ **2-Co2** $\cdots]_{12}$ | na | 28.4 | – |
| **3-Co1** | $[\cdots$ **3-Co1** $\cdots]_{12}$ | na | 25.9 | – |
| **3-Co2** | $[\cdots$ **3-Co2** $\cdots]_{12}$ | 32.4 | 32.9 | – |

## 8 Spin states in dependence on the single-ion anisotropy

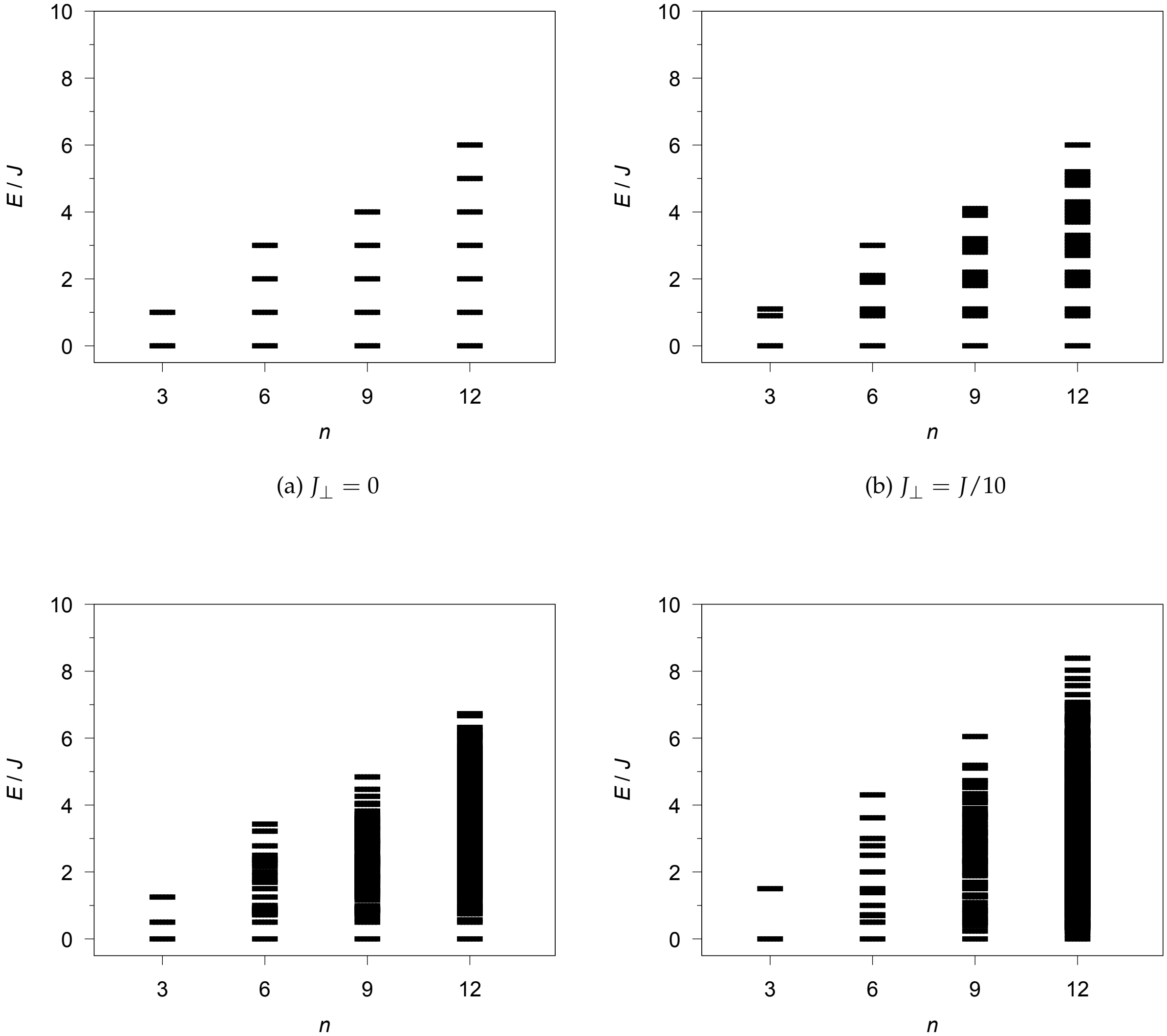


(a) $J_{\perp} = 0$

(b) $J_{\perp} = J/10$

(c) $J_{\perp} = J/2$

(d) $J_{\perp} = J$

**Fig. S13:** Energy spectrum scaled in units of $J$ for an ***n*-membered spin ring** coupling scheme simulating different spin anisotropies (as obtained by (S3) with $J_{\|} \equiv J$): (a) Ising, (b) weak anisotropic Heisenberg, (c) anisotropic Heisenberg, (d) isotropic Heisenberg. The lowering of the anisotropy from (a) to (d) goes together with an overlap of the higher spin multiplets.

$$\begin{aligned}\hat{\mathrm{H}}_{\mathrm{ring}} = &- J_{\|}\left[\mathrm{S}_1^z\,\mathrm{S}_n^z + \sum_{i=1}^{n-1} \mathrm{S}_i^z\,\mathrm{S}_{i+1}^z\right] \\ &- J_{\perp}\left[\mathrm{S}_1^x\,\mathrm{S}_n^x + \mathrm{S}_1^y\,\mathrm{S}_n^y + \sum_{i=1}^{n-1}\left[\mathrm{S}_i^x\,\mathrm{S}_{i+1}^x + \mathrm{S}_i^y\,\mathrm{S}_{i+1}^y\right]\right]\end{aligned} \tag{S3}$$

## 9 Additional POLY_ANISO results

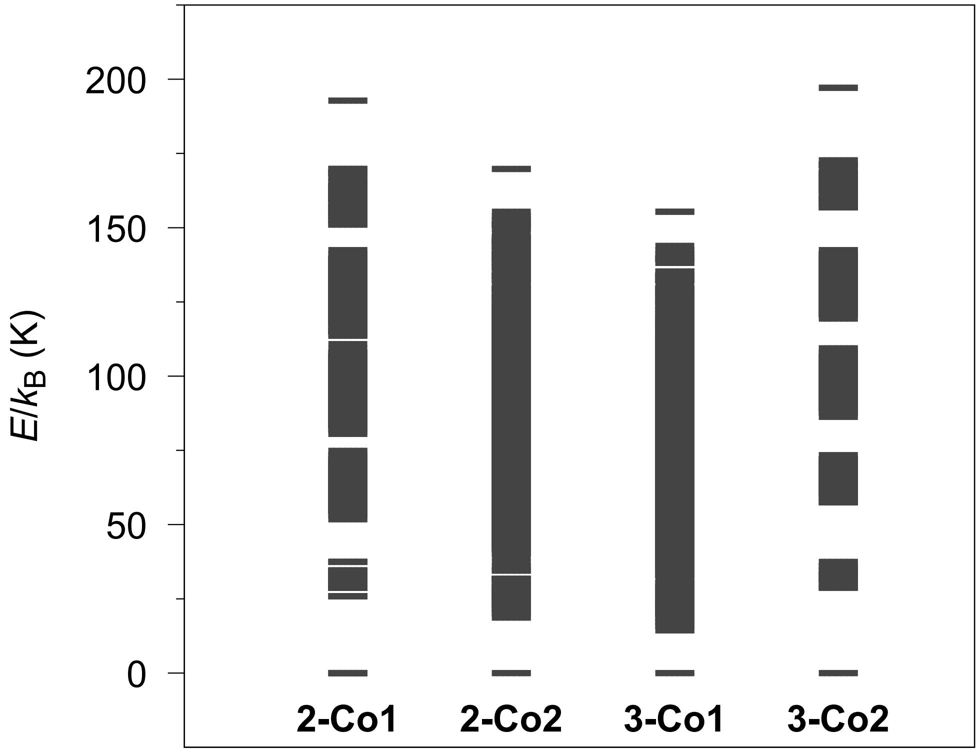


**Fig. S14:** Spin states of hypothetical compounds consisting of only one crystallographically independent spin center (**2-Co1**, **2-Co2**, **3-Co1**, and **3-Co2**) as obtained by the POLY_ANISO program employing the *ab initio* fragment calculations. A 12-membered spin ring coupling scheme together with the fitted theoretical coupling constants $J_{ij}$ have been used. The lower single-ion anisotropy in case of **2-Co2** and **3-Co1** goes together with an overlap of the higher spin multiplets (*cf.* Fig. S13).

**Table S7:** Calculated Cartesian components of the **g** tensor for the ground state doublet in **1**–**3** employing a 12-membered spin ring coupling scheme ($S_{\text{eff}} = 1/2$; $g_{\parallel} = g_z/n$ with $n = 12$).

| | **1** | **2** | **3** |
|---|---|---|---|
| $n$ | 12 | 12 | 12 |
| Simulated system | [··· **1-Co1** ···]$_{12}$ | [··· **2-Co1** ··· **2-Co2** ···]$_6$ | [··· **3-Co1** ··· **3-Co2** ··· **3-Co2** ···]$_4$ |
| $g_x$ | 0.000 | 0.000 | 0.000 |
| $g_y$ | 0.000 | 0.000 | 0.000 |
| $g_z$ | 95.208 | 81.259 | 87.854 |
| $g_{\parallel}$ | 7.934 | 6.772 | 7.321 |

**Table S8:** List of POLY_ANISO simulations employing a coupling scheme of an ***n*-membered spin ring** for **1**–**3** used for the extrapolation of magnetic properties. The simulations were performed in a way that takes the correct repeating sequence and ratio of the crystallographically independent cobalt(II) centers into account.

| $n$ | **1** | **2** | **3** |
|---|---|---|---|
| 3 | [··· **1-Co1** ···]$_3$ | | [··· **3-Co1** ··· **3-Co2** ··· **3-Co2** ···] |
| 4 | [··· **1-Co1** ···]$_4$ | [··· **2-Co1** ··· **2-Co2** ···]$_2$ | |
| 5 | [··· **1-Co1** ···]$_5$ | | |
| 6 | [··· **1-Co1** ···]$_6$ | [··· **2-Co1** ··· **2-Co2** ···]$_3$ | [··· **3-Co1** ··· **3-Co2** ··· **3-Co2** ···]$_2$ |
| 7 | [··· **1-Co1** ···]$_7$ | | |
| 8 | [··· **1-Co1** ···]$_8$ | [··· **2-Co1** ··· **2-Co2** ···]$_4$ | |
| 9 | [··· **1-Co1** ···]$_9$ | | [··· **3-Co1** ··· **3-Co2** ··· **3-Co2** ···]$_3$ |
| 10 | [··· **1-Co1** ···]$_{10}$ | [··· **2-Co1** ··· **2-Co2** ···]$_5$ | |
| 11 | [··· **1-Co1** ···]$_{11}$ | | |
| 12 | [··· **1-Co1** ···]$_{12}$ | [··· **2-Co1** ··· **2-Co2** ···]$_6$ | [··· **3-Co1** ··· **3-Co2** ··· **3-Co2** ···]$_4$ |

## 10 Extrapolation of the magnetic susceptibility

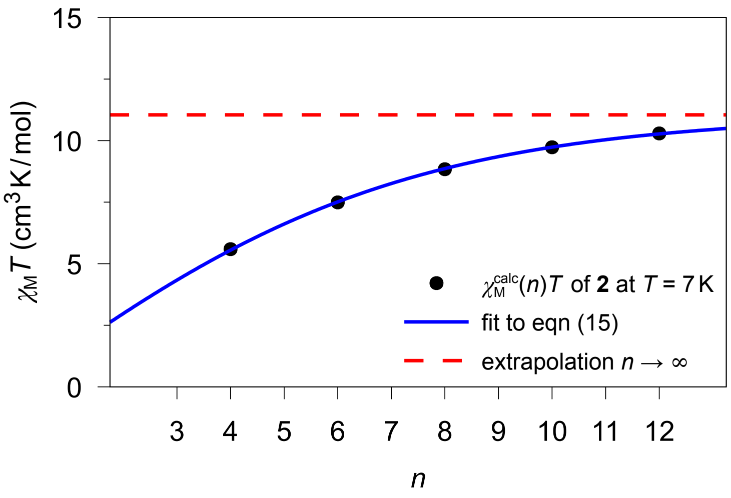


**Fig. S15:** Calculated magnetic susceptibility as $\chi_M^{calc}(n)T$ of **2** at $T = 7\,K$ for different model sizes $n$ of an ***n*-membered spin ring** employing the theoretical coupling constant $J_{12}/k_B = 4.89\,K$. The blue solid line represents the best fit ($a = 11.04(5)\,cm^3K/mol$; $b = 0.7585(20)$) according to eqn (15) from the main manuscript and the red dashed line represents the limit for a periodic system ($n \to \infty$), *i.e.* the parameter $a$.

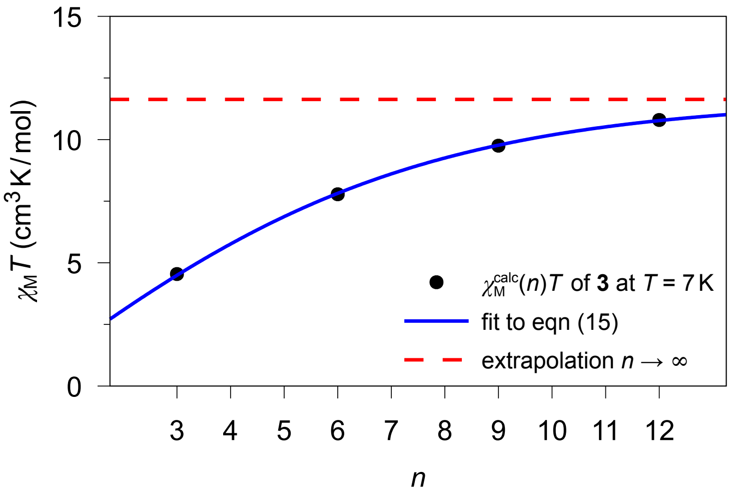


**Fig. S16:** Calculated magnetic susceptibility as $\chi_M^{calc}(n)T$ of **3** at $T = 7\,K$ for different model sizes $n$ of an $n$-MEMBERED SPIN RING employing the theoretical coupling constant $J_{12}/k_B \equiv J_{22}/k_B = 4.82\,K$. The blue solid line represents the best fit ($a = 11.63(8)\,cm^3K/mol$; $b = 0.7621(31)$) according to eqn (15) from the main manuscript and the red dashed line represents the limit for a periodic system ($n \to \infty$), *i.e.* the parameter $a$.

## 11 Magnetic interchain interactions

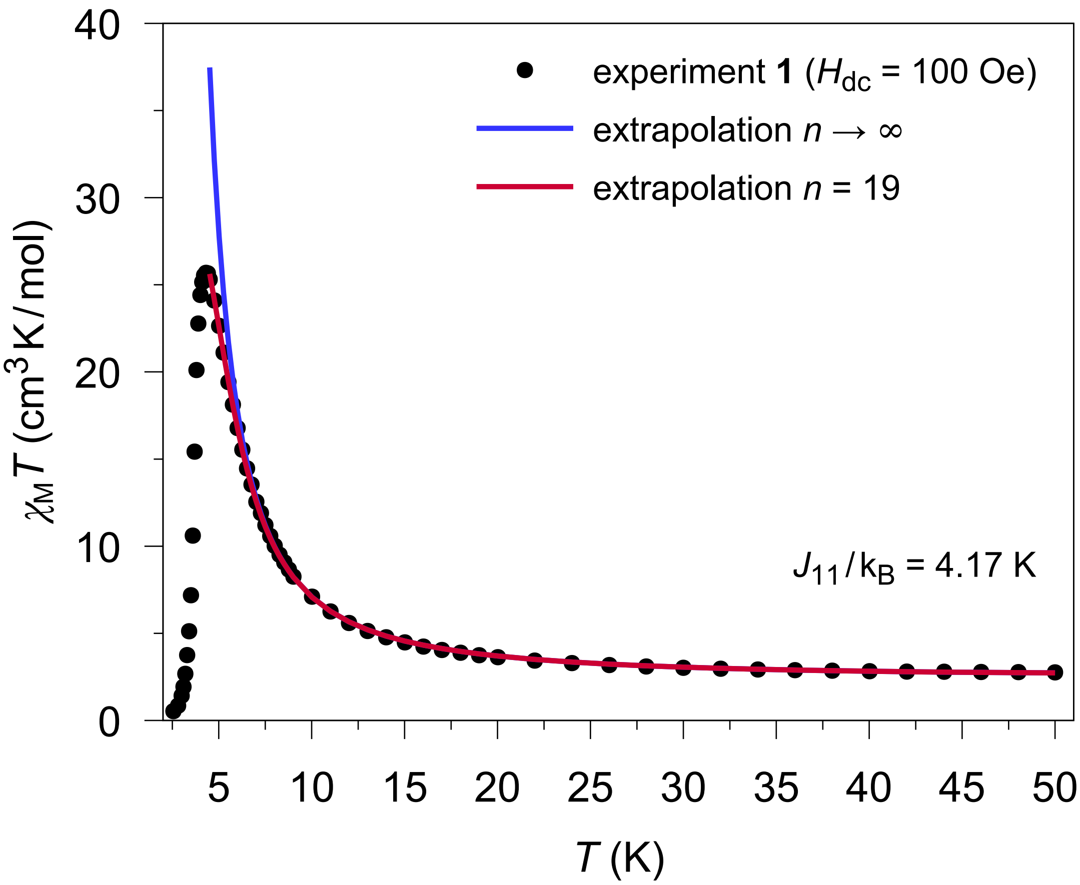


**Fig. S17:** Experimental and simulated temperature dependence of $\chi_M T$ (•) for **1**. Colored lines represent extrapolations of different sizes of the ***n*-membered spin ring** model (red: $n = 19$; blue: $n \to \infty$) based on *ab initio* fragment calculations to investigate the low temperature behavior of the magnetic susceptibility (4.5 K $\leq T \leq$ 50 K).

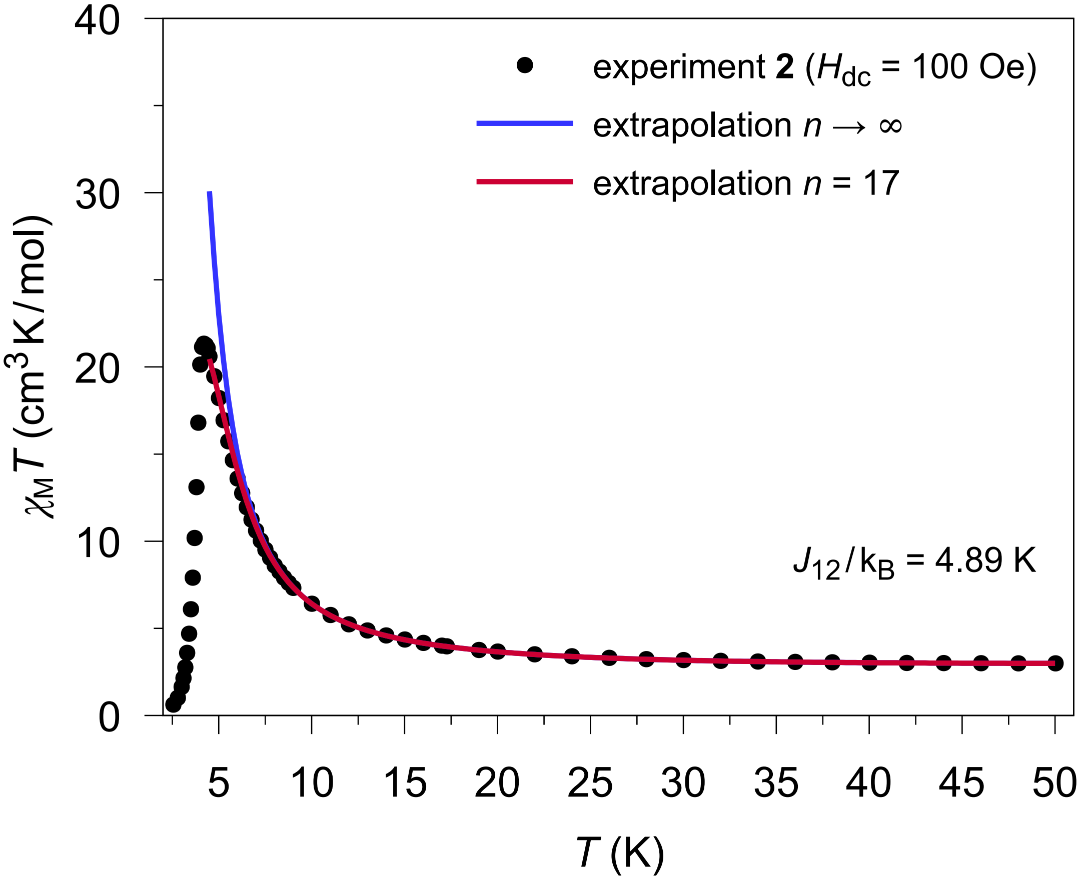


**Fig. S18:** Experimental and simulated temperature dependency of $\chi_M T$ (•) for **2**. Colored lines represent extrapolations of different sizes of the ***n*-membered spin ring** model (red: $n = 17$; blue: $n \to \infty$) based on *ab initio* fragment calculations to investigate the low temperature behavior of the magnetic susceptibility ($4.5\,\mathrm{K} \leq T \leq 50\,\mathrm{K}$).

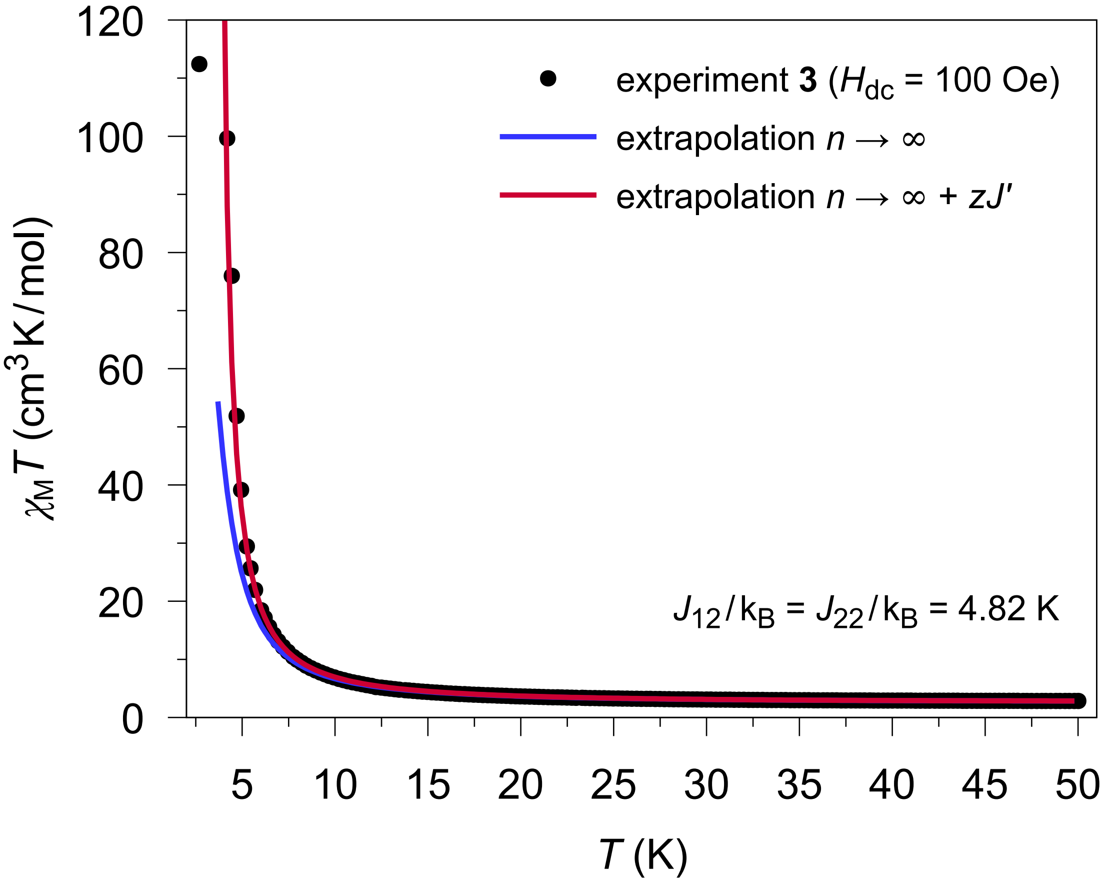


**Fig. S19:** Experimental and simulated temperature dependence of $\chi_M T$ (•) for **3**. The blue solid line represents an extrapolation $n \rightarrow \infty$ of the ***n*-membered spin ring** model based on *ab initio* fragment calculations. The red solid line shows the mean-field corrected magnetic susceptibility according to eqn (16) of the main manuscript ($n \rightarrow \infty$; $zJ'/k_B = 0.85\,K$).

## 12 Basis set information

**Table S9:** Basis sets used for the *ab initio* calculations of **1–3**.

| Atom | Basis set |
|---|---|
| Co | Co.ANO-RCC...6s5p4d2f1g. |
| Zn | Zn.ANO-RCC...5s4p2d. |
| S (donor) | S.ANO-RCC...5s4p2d1f. |
| S (non-donor) | S.ANO-RCC...4s3p. |
| N (donor) | N.ANO-RCC...4s3p2d1f. |
| N (non-donor) | N.ANO-RCC...3s2p. |
| O | O.ANO-RCC...3s2p. |
| C | C.ANO-RCC...3s2p. |
| H | H.ANO-RCC...2s. |

## 13 Decomposition of the calculated magnetic susceptibility

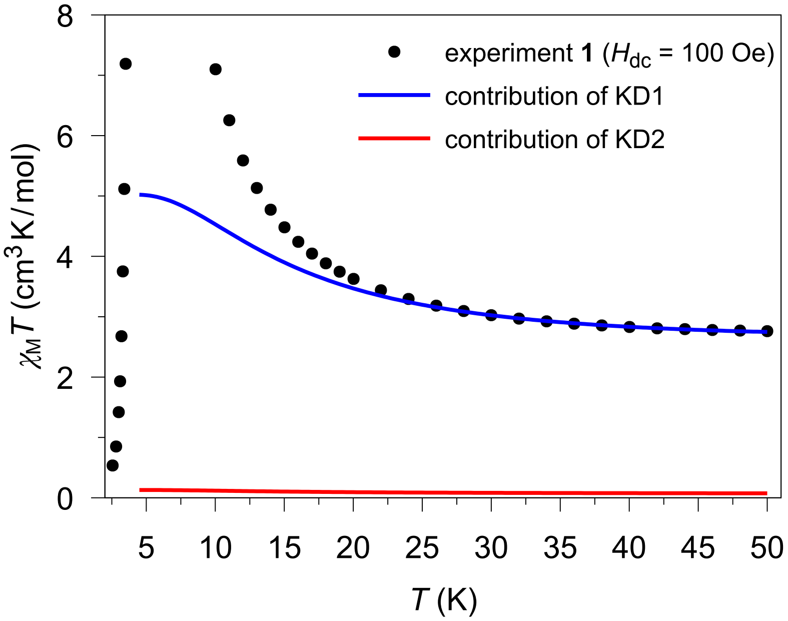


**Fig. S20:** Experimental (•) and calculated magnetic susceptibility $\chi_{\mathrm{M}}^{\mathrm{calc}}(n = 6)T$ of a six-membered spin ring for **1** (colored lines) decomposed into the contribution of the ground state Kramers doublet (blue line) and first excited Kramers doublet (red line), respectively. For this simulation the lowest two KDs were taken into account ($4^6 = 4096$ microstates) instead of the lowest KD ($2^6 = 64$ microstates; $J_{11}/k_{\mathrm{B}} = 4.17\,\mathrm{K}$).

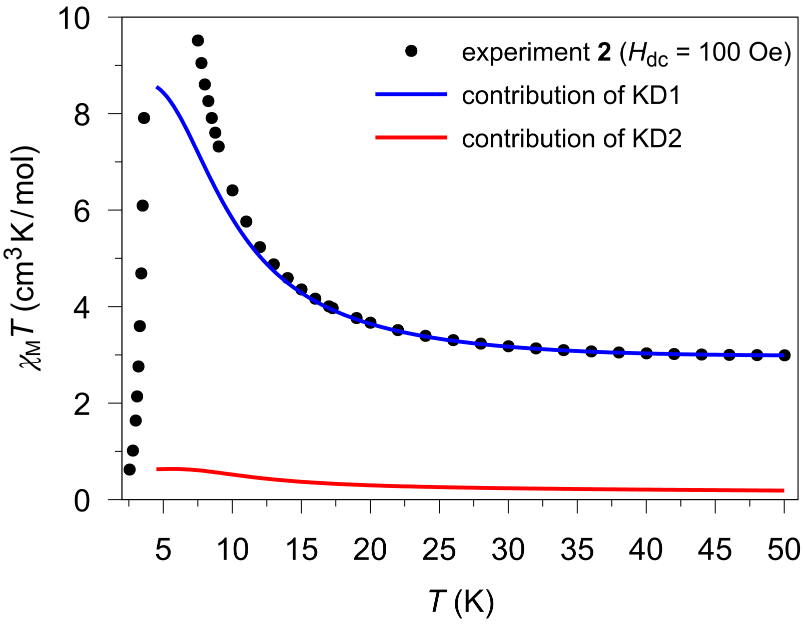


**Fig. S21:** Experimental (•) and calculated magnetic susceptibility $\chi_M^{calc}(n = 6)T$ of a six-membered spin ring for **2** (colored lines) decomposed into the contribution of the ground state Kramers doublet (blue line) and first excited Kramers doublet (red line), respectively. For this simulation the lowest two KDs were taken into account ($4^6 = 4096$ microstates) instead of the lowest KD ($2^6 = 64$ microstates; $J_{12}/k_B = 4.89$ K).

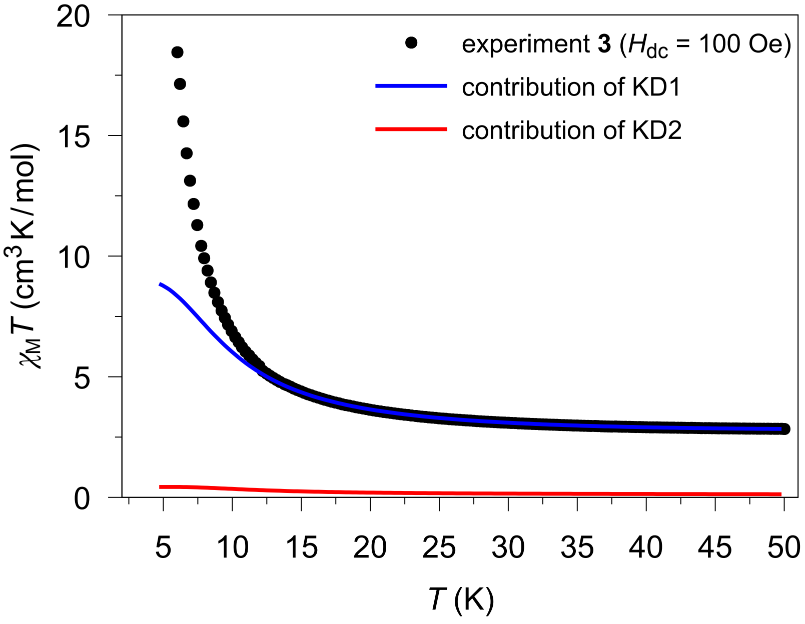


**Fig. S22:** Experimental (•) and calculated magnetic susceptibility $\chi_M^{calc}(n = 6)T$ of a six-membered spin ring for **3** (colored lines) decomposed into the contribution of the ground state Kramers doublet (blue line) and first excited Kramers doublet (red line), respectively. For this simulation the lowest two KDs were taken into account ($4^6 = 4096$ microstates) instead of the lowest KD ($2^6 = 64$ microstates; $J_{12}/k_B \equiv J_{22}/k_B = 4.82$ K).
